# A mutation-based gene set predicts survival benefit after immunotherapy across multiple cancers and reveals the immune response landscape

**DOI:** 10.1186/s13073-022-01024-y

**Published:** 2022-02-24

**Authors:** Junyu Long, Dongxu Wang, Anqiang Wang, Peipei Chen, Yu Lin, Jin Bian, Xu Yang, Mingjun Zheng, Haohai Zhang, Yongchang Zheng, Xinting Sang, Haitao Zhao

**Affiliations:** 1 Department of Liver Surgery, State Key Laboratory of Complex Severe and Rare Diseases, Peking Union Medical College Hospital, Chinese Academy of Medical Sciences and Peking Union Medical College (CAMS & PUMC), Beijing, China; 2 Department of Hepatobiliary Surgery, General Surgery, Qilu Hospital, Cheeloo College of Medicine, Shandong University, Jinan, China; 3 Department of Gastrointestinal Surgery, Key Laboratory of Carcinogenesis and Translational Research, Ministry of Education, Peking University Cancer Hospital & Institute, Beijing, China; 4 Department of Clinical Nutrition and Department of Health Medicine, Peking Union Medical College Hospital, Chinese Academy of Medical Sciences (CAMS) and Peking Union Medical College (PUMC), Beijing, China; 5 Shenzhen Withsum Technology Limited, Shenzhen, China; 6 Department of Obstetrics and Gynecology, University Hospital, LMU Munich, Munich, Germany; 7 Liver Center and The Transplant Institute, Department of Medicine, Beth Israel Deaconess Medical Center, Harvard Medical School, Boston, MA USA

**Keywords:** Tumor microenvironment, Biomarker, Immune response, Immune checkpoint inhibitor, Immunotherapy

## Abstract

**Background:**

Immune checkpoint inhibitor (ICI) therapy has revolutionized the treatment of many cancers. However, the limited population that benefits from ICI therapy makes it necessary to screen predictive biomarkers for stratifying patients. Currently, many biomarkers, such as tumor mutational burden (TMB), have been used in the clinic as indicative biomarkers. However, some high-TMB patients with mutations in genes that are closely related to immunotherapeutic resistance are not sensitive to ICI therapy. Thus, there is a need to move beyond TMB and identify specific genetic determinants of the response to ICI therapy. In this study, we established a comprehensive mutation-based gene set across different tumor types to predict the efficacy of ICI therapy.

**Methods:**

We constructed and validated a mutational signature to predict the prognosis of patients treated with ICI therapy. Then, the underlying immune response landscapes of different subtypes were investigated with multidimensional data.

**Results:**

This study included genomic and clinical data for 12,647 patients. An eleven-gene mutation-based gene set was generated to divide patients into a high-risk group and a low-risk group in a training cohort (1572 patients with 9 types of cancers who were treated with ICI therapy). Validation was performed in a validation cohort (932 patients with 5 types of cancers who were treated with ICI therapy). Mutations in these 11 genes were associated with a better response to ICI therapy. In addition, the mutation-based gene set was demonstrated to be an independent prognostic factor after ICI therapy. We further explored the role of the immune context in determining the benefits of immunotherapy in 10,143 patients with 33 types of cancers and found distinct immune landscapes for the high- and low-risk groups.

**Conclusions:**

The mutation-based gene set developed in this study can be used to reliably predict survival benefit across cancers in patients receiving ICI therapy. The close interplay between the extrinsic and intrinsic immune landscapes in the identified patient subgroups and the subgroups’ differing responses to ICI therapy could guide immunotherapy treatment decisions for cancer patients.

**Supplementary Information:**

The online version contains supplementary material available at 10.1186/s13073-022-01024-y.

## Background

The treatment landscapes of different cancer types have changed based on developments in the field of immuno-oncology. Immune checkpoint inhibitor (ICI) therapy, which includes antibodies targeting cytotoxic T lymphocyte-associated protein-4 (CTLA-4), programmed death-1 (PD-1), and programmed death ligand-1 (PD-L1), offers significant clinical benefits for patients with many types of cancer [1–3]. In the PD-1/PD-L1 pathway, binding of the PD-1 receptor on T cells to PD-L1 on antigen-presenting cells and tumor cells limits or halts the T cell response by downregulating cytokine production, effector function, and T cell proliferation. However, only 20–40% of patients treated with PD-1/PD-L1 blockade therapy show a response, whereas most do not, and the determinants of the response remain elusive. In addition, treatment discontinuation in nonresponders is often delayed due to difficulty in interpreting imaging results [4]. Therefore, the selection of patients is vital, and it is a substantial challenge to identify reliable markers to rapidly predict a sustainable response.

Tumor-infiltrating immune cells play a crucial role in patient prognosis and cancer treatment efficacy [5–8]. In the tumor microenvironment, the composition of immune cells is related to cancer heterogeneity and creates complexity that is interesting but challenging when studying the dynamic interactions between cancer and immune cells [8]. IFN-γ is a crucial cytokine produced by natural killer (NK) cells and activated T cells [9], and loss of sensitivity to IFN-γ induction can result in resistance to immunotherapy [6, 10]. Many excellent models and targets for predicting the response to ICI therapy have been developed recently. For example, Jiang et al. developed a tumor immune dysfunction and exclusion (TIDE) score, a method that uses gene expression profiles for calculations to predict the response to ICI therapy. TIDE evaluates two different tumor immune escape mechanisms: the prevention of T cell infiltration in cancers with low cytotoxic T lymphocyte (CTL) levels and the induction of T cell dysfunction in cancers with high CTL infiltration [10, 11]. This method can predict the outcomes of cancer patients treated with ICI therapy more accurately than other biomarkers, such as mutational load and PD-L1 levels. Moreover, Shi et al. discovered that MAN2A1 loss renders cancer cells more susceptible to T cell-mediated killing and that inhibition of MAN2A1 enhances the immune response to anti-PD-L1 [12]. In addition to transcriptomics data, other types of omics data can be assessed to predict the efficacy of ICI therapy. Kumar et al. found that suppression of CARM1, an epigenetic enzyme and cotranscriptional activator, facilitates immunotherapy for resistant tumors through dual effects on cancer cells and cytotoxic T cells [13]. Genomic profiling also represents an emerging approach for predicting the response to immunotherapy. Based on exome analysis of tumors from pembrolizumab-treated patients, the best responses to PD-1 blockade occurred in tumors with a high tumor mutational burden (TMB) [14]. Indeed, TMB has been shown to be a strong marker of the response to front-line treatment with nivolumab together with ipilimumab in patients with advanced non-small-cell lung cancer (NSCLC) [15]. However, due to the limited number of high-quality DNA samples, the availability of tissue samples, the need for bioinformatics analyses, the lack of a standardized panel and cutoff values, and the high cost, it is difficult to implement whole-exome sequencing or next-generation sequencing panels in routine clinical practice.

Furthermore, tumors with a comparably high TMB show variable responses, indicating that additional factors may contribute to the response to ICIs [16]. Mutations in genes involved in antigen presentation and in interferon-receptor signaling pathways, such as B2M and JAK1/2, have been shown to be related to acquired resistance to ICIs, and JAK1/2 mutations have also been found to result in primary anti-PD-1 resistance [17]. Although the TMB may be high, patients carrying these mutations usually have a poor response to ICIs. There is thus a need to move beyond the TMB and identify specific genetic determinants of the response to PD-1 inhibitors [18].

POLE and POLD1 mutations have been proposed as biomarkers for immunotherapy outcomes across multiple cancer types [19]. However, there has not yet been a comprehensive exploration of factors related to prognosis after immunotherapy at the genomic level. In this study, we conducted a pancancer genomic analysis to identify a powerful signature for predicting the clinical benefit for ICI-treated patients. We further investigated the role of the immune context in determining the benefit of immunotherapy.

## Methods

### Study population

Mutation data and clinical information for the training and validation cohorts were obtained from the cBioPortal database (https://www.cbioportal.org) and the literature [20–28]. The predictive model was first constructed based on the training cohort, which consisted of 1572 patients with 9 types of cancers who received ICI treatment (Additional file 2: Fig. S1) and was then validated in the independent validation cohort consisting of 932 patients with 5 types of cancers who received ICI treatment (Additional file 2: Fig. S1) [20, 23]. Additional file 2: Fig. S1 summarizes the sample selection process. Specifically, in the training cohort from Samstein et al. [20], both mutation profiles and clinical data were available for 1661 patients. Next, cancer types with only one case (*n* = 1) and cancer of unknown primary type (*n* = 88) were excluded; 1572 cases remained. In the validation cohort, both mutation profiles and clinical data were available for 144 patients from the cohort of Liu et al. [21], 274 patients from the IMvigor210 cohort reported by Mariathasan et al. [22], 249 patients from the cohort of Miao et al. [23], 35 patients from the cohort of Miao et al. [24], 68 patients from the cohort of Riaz et al. [25], 38 patients from the cohort of Hugo et al. [26], 110 patients from the cohort of Van Allen et al. [27], and 64 patients from the cohort of Snyder et al. [29]. Notably, in the Miao cohort (*n* = 249) [23], cancer types with only one case (*n* = 3) were excluded, with only 246 cases remaining. In the Hugo cohort (*n* = 38) [26], one patient was excluded because of a lack of overall survival data; 37 cases remained. In addition, 46 cases from the Snyder cohort (*n* = 64) and Miao cohort (*n* = 249) were duplicates, and 46 cases from the Snyder cohort were excluded [23, 29]. The clinical data for each sample used in the analysis are shown in Additional file 1: Tables S1-S3.

Samples from the training cohort were sequenced using the Memorial Sloan Kettering-Integrated Mutation Profiling of Actionable Cancer Targets (MSK-IMPACT) panel, which was designed for targeted sequencing of 468 tumor-suppressor genes, oncogenes, and members of pathways considered actionable for targeted therapies and authorized by the US FDA [20]. Samples from the validation cohort were sequenced using WES, except for the IMvigor210 cohort reported by Mariathasan et al., which was sequenced with the FoundationOne panel, a US FDA-authorized panel [22]. This study included all nonsynonymous mutations, including missense, frame-shift, nonsense, nonstop, splice site, and translation start site mutations [19]. The primary clinical outcomes were overall survival (OS) and clinical benefit, which was categorized as durable clinical benefit (DCB) (complete response [CR]/partial response [PR] or stable disease [SD] that lasted > 6 months) or no durable benefit (NDB) (progression of disease [PD] or SD that lasted ≤ 6 months) [30]. In the training cohort, no response data were provided by Samstein et al., and we extracted the response data for some of those patients from Janjigian et al. [31] and Rizvi et al. [30]. In the validation cohort, data on the response to ICI therapy were obtained from Hugo et al. [26], Liu et al. [21], Mariathasan et al. [22], Miao et al. [23, 24], Riaz et al. [25], and Van Allen et al. [27]. In the training and validation cohorts, OS was defined as the time from the date of the first ICI therapy to the time of the last follow-up or death. For samples sequenced by WES, the TMB was defined as the total number of nonsynonymous mutations divided by the exome size (38 Mb was utilized as the exome size). For samples sequenced with the MSK-IMPACT panel or FoundationOne panel, the TMB was obtained from the respective studies.

In the cohort from TCGA, mutation profiles (sequenced by WES), copy number variation (CNV) data, and mRNA expression profiles for 10143 patients with 33 cancer types, as acquired from the PanCancer Atlas consortium (https://gdc.cancer.gov/about-data/publications/pancanatlas), were employed to explore differences in genomic patterns between the identified subtypes [32].

### Propensity score matching (PSM) weighting algorithm

PSM is a critical statistical method used to adjust for confounding factors in observational studies and has a wide range of applications in the social sciences, economics, and clinical practice [33]. In contrast to pair matching, PSM can improve balance, estimate efficiency, and enable the inclusion of all subjects by weighting them such that each contributes to the estimation [34]. We used the PSM method in this study to balance potentially confounding factors, including age, drug type, and cancer type, between the mutant and wild-type status of each gene in the MSK-IMPACT panel. Briefly, we first calculated the propensity score using logistic regression, with the mutation status of a given gene as a dependent variable, and we then used the PSM weighting scheme to continuously assign weights for each sample based on the propensity scores to achieve balance [34]. When the standardized difference of the weighted propensity scores between the mutant gene and wild-type gene groups was less than 10%, we considered the clinical characteristics to be balanced between the propensity score-weighted samples. We then compared survival data between the mutant gene and wild-type gene samples by supplying weights for multivariate Cox regression. Genes with a *P* value < 0.05 and adjusted *P* < 0.1 were considered to have a profound effect on prognosis and were selected for further analysis, and statistical significance was confirmed by randomly shuffling the mutation labels of the samples and repeating the above processes 100 times [34]. Statistical significance was analyzed by comparing the number of significant features obtained from the permutated data to that obtained from our real-world data.

### Generation and validation of the mutation-based gene set

In the training cohort, PSM analysis, Lasso-penalized Cox regression analysis, and multivariate Cox regression analysis were employed to screen prognostic genes and construct a mutation-based gene set. First, a gene was considered significant when the *P* value was < 0.05 in the PSM analysis. The PSM algorithm was utilized as described above. Second, we applied Lasso-penalized Cox regression using the “glmnet” R package (version: 4.0-2) to avoid overfitting, reduce multicollinearity, and further select the key prognostic genes [35, 36]; subselection of prognostic genes was performed by shrinkage of the regression coefficient via the imposition of a penalty proportional to size [37]. Tenfold cross-validations were performed to define the optimal value of the lambda penalty parameter; this resulted in the weight of most of the potential prognostic genes decreasing to zero, and a relatively small number of prognostic genes with a weight of nonzero remained. For the Lasso-penalized Cox regression analysis, we subsampled the dataset with replacement 1000 times and selected prognostic genes with nonzero occurrence frequencies of more than 990 [38]. Third, multivariate Cox regression analysis was used to construct a mutation-based gene set with the “survival” R package (version: 3.2-3). The risk score can be estimated from the Cox model as follows:

$$\mathrm{Risk}\ \mathrm{score}=\exp \left[\sum_{\mathrm{i}=1}^{\mathrm{p}}{\mathrm{b}}_{\mathrm{i}}{\mathrm{X}}_{\mathrm{i}}-\sum_{\mathrm{i}=1}^{\mathrm{p}}{\mathrm{b}}_{\mathrm{i}}{\overline{\mathrm{X}}}_{\mathrm{i}}\right]$$

- The coefficients (*b*_1_, *b*_2_, …, *b*_p_) measure the impact (i.e., the effect size) of covariates.
- X_i_ is the value of the ith covariate from the subjects.
- ${\overline{\mathrm{X}}}_{\mathrm{i}}$ is the mean value of the *i*th covariate.

X-tile 3.6.1 software was used to determine the best cutoff for classifying patients into low- and high-risk score groups [39]. The cutoff was defined as the risk score that generated the largest value of χ^2^ in the Mantel–Cox test [40]. Finally, the same formula and cutoff were applied for the validation and TCGA cohorts.

### Generation and validation of the nomogram

As convenient and reliable tools, nomograms are widely used to predict specific outcomes in clinical oncology; they quantitatively predict prognosis for certain patients using known critical predictive factors and reveal the survival probability of clinical outcomes [41]. A calibration curve was used to evaluate the agreement between the actual and predicted survival probabilities [42].

### Evaluation of immune infiltration with CIBERSORT

CIBERSORT is a deconvolution algorithm that is based on gene expression and applies support vector regression to infer cell type proportions in data from bulk cancer samples of mixed cell types [43]. The proportions of 22 types of infiltrating immune cells were estimated via the CIBERSORT method based on normalized gene expression data. CIBERSORT immune infiltration proportions were obtained from the pancancer immune landscape project conducted by Thorsson et al. [44].

### TIL fraction, leukocyte fraction and lymphocyte fraction analyses

In the cohort from TCGA, the levels of TILs from genomics evaluation and those of TILs from H&E-stained image evaluation were evaluated by analyzing the data from Thorsson et al. and Saltz et al., respectively [44, 45]. Saltz et al. presented global mappings of TILs for over 5000 H&E-stained diagnostic whole-slide images from TCGA by using deep learning-based lymphocyte classification with convolutional neural networks (CNNs), representing a benchmark for TIL analysis. Genomics evaluation of the TIL fraction was carried out by multiplying an aggregated proportion of the lymphocyte fraction in the immune compartment assessed by the CIBERSORT approach with the leukocyte fraction derived from DNA methylation. The lymphocyte fraction is an aggregation of CIBERSORT estimates of T regulatory cells, follicular helper T cells, naïve, resting and activated memory CD4 T cells, naïve and memory B cells, plasma cells, activated and resting NK cells, CD8 T cells, and gamma-delta T cells.

### The immune infiltration scores from Danaher et al.

The immune infiltration scores were extracted from a previous TCGA pancancer study conducted by Danaher et al. [46]. Each immune cell score was estimated by 60 specific marker genes with expression levels that are able to classify 14 immune cell populations: total TILs, B cells, DCs, macrophages, exhausted CD8 T cells, CD8 T cells, neutrophils, cytotoxic cells, Tregs, NK CD56dim cells, mast cells, NK cells, and Th1 cells. These results were highly reproducible and concordant with those obtained by immunohistochemistry and flow cytometry.

### Immune signature evaluation

Twenty-nine classical immune signatures were acquired from He et al. (Additional file 1: Table S4) [47]. We used the “GSVA” R package (version: 1.34.0) based on the single-sample gene set enrichment analysis (ssGSEA) method to quantify the enrichment levels of the twenty-nine immune signatures in each sample [48].

### Immunogenomic indicator calculation

Immunogenomic indicators were obtained from the pancancer immune landscape project conducted by Thorsson et al. [44]. In brief, the intertumoral heterogeneity (ITH) score was defined as the subclonal genome fraction (which measures the fraction of the tumor genome that is not part of the “plurality” clone), as determined by ABSOLUTE, which models tumor copy number alterations and mutations as mixtures of subclonal and clonal components of varying ploidy. The copy number burden scores n_segs and frac_altered (“number of segments” and “fraction altered”, respectively) represent the total number of segments in each sample’s copy number profile and the fraction of bases that deviate from the baseline ploidy, respectively. Aneuploidy scores were defined as the sum total of the amplified or deleted (collectively, “altered”) arms. TCR diversity scores (Shannon entropy and richness) and BCR diversity scores (Shannon entropy and richness) were inferred from cancer RNA-seq data.

### Cytolytic activity score

The cytolytic activity score (CYT) was defined as the geometric mean of granzyme A (GZMA) and perforin 1 (PRF1) expression [49].

### Deciphering mutational signatures in the genome

The “MutationalPatterns” R package (version: 1.12.0) was applied to perform nonnegative matrix factorization (NMF) analysis of mutations stratified by 96 trinucleotide contexts in pancancer specimens from TCGA. The extracted mutational portrait was compared against the Catalogue of Somatic Mutations in Cancer (COSMIC) by cosine similarity.

### Enrichment scores of oncogenic pathways

Ten canonical oncogenic pathways containing 187 oncogenes were obtained from the study conducted by Sanchez-Vega et al. [50]. Enrichment scores for each pathway in each sample were determined by the ssGSEA approach applying the “GSVA” R package [48].

### Copy number variation analysis

Significant deletion or amplification events in regions of the genome were investigated with GISTIC 2.0, a revised computational program that identifies somatic copy number alterations by investigating the amplitude and frequency of observed events [51].

### Functional enrichment analysis

Functional enrichment analysis and clustering of the identified biological processes were conducted using the “clusterProfiler” R package (version: 3.14.3) [52].

### Statistical analysis

Associations between the mutation-based gene set and OS were analyzed via the Kaplan–Meier method; survival curves were compared via the log-rank test. C-indexes were determined to compare the accuracy of the mutation-based gene set with that of the risk factors [53]. Statistical analysis for comparisons between two groups was conducted using the Wilcoxon test. R software (version 3.6.3) was applied to perform all statistical analyses, and *P* values were two-tailed. A *P* value < 0.05 was considered to indicate significance.

## Results

### Identification of a mutation-based gene set for predicting immunotherapy outcomes

**Fig. 1:**
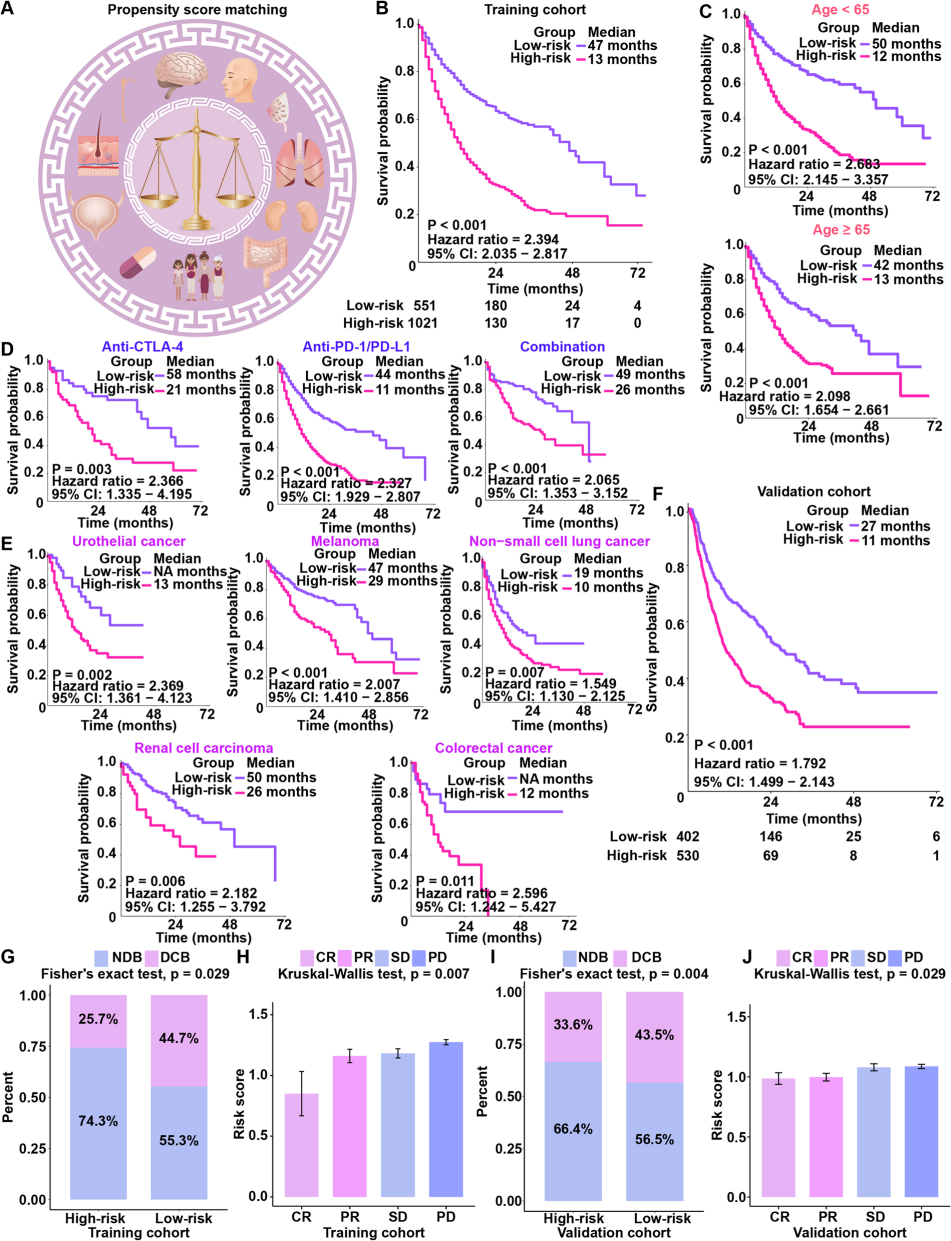
Generation and validation of the mutation-based gene set. **A** An overview of the propensity score algorithm used to balance confounding factors, including age, cancer types, and drug types. **B** Survival analysis of the mutation-based gene set in the training cohort. **C** Survival analysis of the mutation-based gene set in different age groups (age < 65 and age ≥ 65). **D** Survival analysis of the mutation-based gene set in different drug type subgroups (anti-PD-1, anti-CTLA-4 and combination). **E** Survival analysis of the mutation-based gene set in different cancer type subgroups (urothelial cancer, melanoma, non-small-cell lung cancer, renal cell carcinoma, and colorectal cancer). **F** Survival analysis of the mutation-based gene set in the validation cohort. **G** The proportion of patients who responded to ICI therapy in the high-risk and low-risk groups in the training cohort. **H** The distribution of risk scores in groups with different ICI clinical response statuses in the training cohort. **I** The proportions of patients with a response to ICI therapy in the high-risk and low-risk groups in the validation cohort. **J** The distribution of risk scores in groups with different ICI clinical response statuses in the validation cohort

Samples from the training cohort were sequenced using an MSK-IMPACT panel including 468 genes [20]. To remove confounding effects (including effects of age, drug type and cancer type), a PSM weighting algorithm was adopted to study survival differences between carriers of mutant and wild-type variants of these 468 genes (Fig. 1A). Additional file 2: Fig. S2 summarizes the analysis process used for this study. We calculated the propensity scores, “reweighted” the samples in the training cohort, and compared the survival differences between mutant and wild-type status for the 468 genes. As a result, 98 gene mutations were found to be significantly related to OS (*P* < 0.05 and adjusted *P* < 0.1). Lasso-penalized Cox regression analysis was used to further select important genes. Eleven genes with a nonzero occurrence frequency of more than 990 times of a total of 1000 repetitions were obtained (Additional file 1: Table S5) [38]. Finally, we quantified a risk score for each patient on the basis of the eleven-gene mutation-based gene set through multivariate Cox regression analysis:

$$\mathrm{Risk}\ \mathrm{score}=\exp \left[\left(-0.4885006\times \mathrm{BRAF}\right)+\left(-0.2618274\times \mathrm{PAK}7\right)+\left(-0.2610592\times \mathrm{PTPRD}\right)+\left(-0.2404202\times \mathrm{PTPRT}\right)+\left(-0.2321493\times \mathrm{ROS}1\right)+\left(-0.2759073\times \mathrm{SETD}2\right)+\left(-0.8026092\times \mathrm{TET}1\right)+\left(-1.0449158\times \mathrm{VHL}\right)+\left(-1.7929573\times \mathrm{FAM}46\mathrm{C}\right)+\left(-0.7964559\times \mathrm{RNF}43\right)+\left(-0.3821696\times \mathrm{ZFHX}3\right)-\left(-0.3283004\right)\right].$$

In this formula, exp denotes exponential, the mutant gene status equals 1, and the wild-type gene status equals 0. X-tile software was used to generate an optimal cutoff value (1.07) to divide patients into groups with high- and low-risk scores [40]. The cutoff score of 1.07 was automatically identified by X-tile software because it was defined as the risk score that generated the largest value of *χ*^2^ in the Mantel–Cox test. In addition, we rescored 9 gene sets (excluding ROS1 and PTPRT from the 11 gene sets), and we found that the cutoff automatically identified by X-tile software was 0.74, so the cutoff for the 9 gene sets was 0.74, and the cutoff for the 11 gene sets was 1.07. This means that the 1.07 cutoff used in our study was selected specifically for 11 gene sets.

Patients in the group with high risk scores had a shorter OS than those in the group with low risk scores (*P* < 0.001; HR, 2.394; 95% CI, 2.035–2.817) (Fig. 1B). The AUC of the mutation-based gene set in the training cohort was 0.751 at 3 years and 0.831 at 5 years (Additional file 2: Fig. S3A). The AUC of each cancer type in the training cohort was also calculated (Additional file 2: Fig. S3C). We investigated whether the mutation-based gene set is restricted to specific groups or applicable to different populations. Subgroup analyses indicated that the mutation-based gene set was significantly associated with OS in patients treated with ICI therapy, regardless of age (Fig. 1C), drug type (Fig. 1D), or cancer type (Fig. 1E). The results of the subgroup analysis are in good agreement with those of PSM. Considering that TMB is a good marker for predicting the efficacy of immunotherapy, we performed a stratified analysis of TMB [54, 55]. In the stratified analysis of TMB, we found that the mutation-based gene set could predict prognosis very well in the TMB-high group and TMB-low group (Additional file 2: Fig. S4A and B). In the training cohort, the survival time of patients with BRAF mutation (median OS: 47.0 months) was significantly longer than that of those with wild-type BRAF (median OS: 17.0 months) (*P* < 0.001) (Additional file 2: Fig. S4C). In melanoma, patients with mutated BRAF (median OS: 49.0 months) had a good survival trend compared with those with wild-type BRAF (median OS: 33.0 months) (Additional file 2: Fig. S4D). We calculated the mutation rate of the ROS1 (Additional file 2: Fig. S4E) and PTPRT (Additional file 2: Fig. S4F) genes for each cancer type and found them not to be high.

### Validation of the mutation-based gene set for predicting immunotherapy outcomes

To further confirm the value of the mutation-based gene set for predicting immunotherapy outcomes, we evaluated the mutation-based gene set in the validation cohort. When using the same formula and the same cutoff obtained from the training cohort, in the validation cohort, patients in the low-risk group exhibited an increased OS compared with those in the high-risk group (*P* < 0.001; HR, 1.792, 95% CI, 1.499–2.143) (Fig. 1F). The AUC of the mutation-based gene set in the validation cohort was 0.674 at 3 years and 0.732 at 5 years (Additional file 2: Fig. S3B); the AUC for each cancer type in the validation cohort was also assessed (Additional file 2: Fig. S3D). Considering the dependence of TMB measurement on the sequence panels used [54, 55], we separately evaluated the robustness of the model across different panels used in the clinic. In the Snyder et al. cohort [29], an advanced melanoma anti-CTLA-4-treated cohort (Additional file 2: Fig. S5A), and in the Mariathasan et al. cohort [22], a metastatic urothelial cancer anti-PD-L1-treated cohort (Additional file 2: Fig. S5B), the survival time of patients in the low-risk group was significantly longer than that of patients in the high-risk group (*P* < 0.05), which was consistent with the results obtained for the training cohort.

We also systematically compared the performance of our mutation-based gene set to that of the existing mutation-based signature of ICI response in the training cohort, including frameshift insertion/deletion (indel) mutation burden [56], tobacco mutation signature [57], UV signature [58], APOBEC signature [59], and DNA damage response pathway mutation [60]. Genes of the DNA damage response pathway were extracted from Conway et al., including MSH2, MSH6, PMS2, POLE, and BRCA2 [60]. We defined the sample in which all genes in the DNA damage response pathway were wild-type as “DNA damage response pathway unaltered” and the sample in which at least one gene in the DNA damage response pathway was mutated as “DNA damage response pathway altered.” The C-index is one of the most commonly used performance measures for survival models: the higher the value of the C-index is, the better the predictive ability of the model [61]. We found that the predictive power of the mutation-based gene set (C-index = 0.716) was greater than that of the frameshift insertion/deletion (indel) mutation burden (C-index = 0.526), tobacco mutation signature (C-index = 0.515), UV signature (C-index = 0.592), APOBEC signature (C-index = 0.531), and DNA damage response pathway mutation (C-index = 0.607) (Additional file 2: Fig. S5C).

As many studies have associated individual gene mutation status with ICI benefit, we used the C-index to compare the performance of mutation-based gene sets to that of those genes, including B2M [62], JAK1, JAK2 [63], KRAS, TP53 [64], PTEN [65], STK11 [66], and BAP1 [67]. We found that the predictive power of the mutation-based gene set (C-index = 0.716) was greater than that of B2M mutation (C-index = 0.538), JAK1 mutation (C-index = 0.614), JAK2 mutation (C-index = 0.615), KRAS mutation (C-index = 0.526), TP53 mutation (C-index = 0.600), PTEN mutation (C-index = 0.513), STK11 mutation (C-index = 0.653), and BAP1 mutation (C-index = 0.606) (Additional file 2: Fig. S5D).

We investigated whether the mutation-based gene set is able to predict the response to ICI therapy in the training and validation cohorts. In the training cohort, there was a significant DCB of ICI therapy in the low-risk group compared to the high-risk group (Fig. 1G). Patients with low risk scores were also more likely to respond to ICI therapy (Fig. 1H). This result was confirmed in the validation cohort (Fig. 1I and J). Additionally, we examined the breakdown of the risk score-predicted high-risk and low-risk proportion per cancer type and found that renal cell carcinoma and melanoma accounted for a higher proportion of samples in the low-risk group (Additional file 2: Fig. S6A-C). This may be due to the higher response rate of renal cell carcinoma and melanoma than other tumors to immunotherapy [68].

### The mutation-based gene set is an independent predictor of prognosis after immunotherapy

**Fig. 2:**
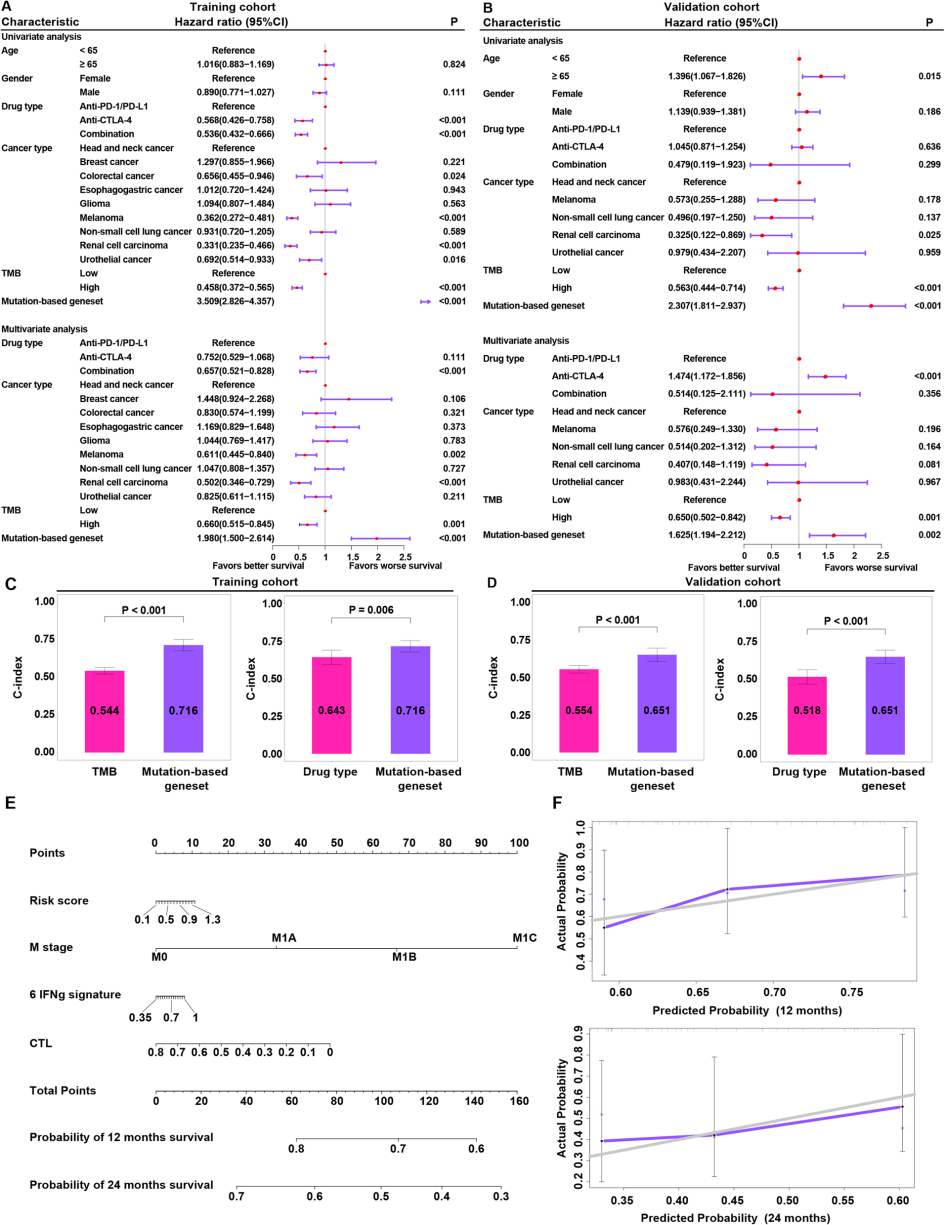
Relationships between the mutation-based gene set and other characteristics. **A** Univariate and multivariate Cox regression analyses of the mutation-based gene set in the training cohort. **B** Univariate and multivariate Cox regression analyses of the mutation-based gene set in the validation cohort. **C** Comparison of C-indexes between the mutation-based gene set and the TMB and drug type in the training cohort. Error bars represent the 95% CI of the C-index. **D** Comparison of C-indexes between the mutation-based gene set and the TMB and drug type in the validation cohort. **E** Nomogram for predicting survival probability at 12 and 24 months in the Riaz cohort. **F** Calibration curves for evaluating the predictive accuracy of the nomogram in the Riaz cohort. The gray line represents ideal performance. The purple line represents actual performance

We next verified whether the mutation-based gene set is an independent predictor of the response to immunotherapy. In both the training cohort and validation cohorts, univariate Cox regression analysis showed that the mutation-based gene set correlated with OS (Fig. 2A, B). After adjusting for drug type, cancer type, and TMB, the mutation-based gene set remained an independent predictive factor based on multivariate Cox regression analysis, confirming its robustness for independently predicting ICI prognosis (Fig. 2A, B).

In both the training and validation cohorts, multivariate Cox regression analysis showed drug type, TMB, and the mutation-based gene set to be independent predictive factors for identifying patients who will benefit from ICI treatment (Fig. 2A, B). To identify which factor has the best predictive performance, the C-index was utilized to compare performance between the mutation-based gene set and the TMB and drug type in both the training and validation cohorts. In the former, the C-index results showed that the mutation-based gene set predicted prognosis more accurately than TMB (*P* < 0.001) and drug type (*P* = 0.006) (Fig. 2C), a result that was validated in the validation cohort (all *P* < 0.001) (Fig. 2D).

### The mutation-based gene set, disease stage, CTL, and 6-IFN-g gene signature can be combined to predict the clinical benefit of ICI therapy

Using the Riaz cohort involving both DNA sequencing and RNA sequencing data [25], we compared the 2-gene cytolytic score, 6-gene IFN-g signature score, and 18-gene IFN-g signature score between the low-risk group and the high-risk group. The genes of the 2-gene cytolytic score included GZMA and GZMB. The genes of the 6-gene IFN-g signature were extracted from Ayers et al.: IDO1, CXCL10, CXCL9, HLA-DRA, STAT1, and IFNG [9]. The genes of the 18-gene IFN-g signature were extracted from Ayers et al.: CD3D, IDO1, CIITA, CD3E, CCL5, GZMK, CD2, HLA-DRA, CXCL13, IL2RG, NKG7, HLA-E, CXCR6, LAG3, TAGAP, CXCL10, STAT1, and GZMB [9]. The 2-gene cytolytic score, 6-gene IFN-g signature score, and 18-gene IFN-g signature score were estimated by the ssGSEA method. We found that compared with the high-risk group, the low-risk group showed a higher 2-gene cytolytic score (*P* = 0.071), 6-gene IFN-g signature score (*P* < 0.05), and 18-gene IFN-g signature score (*P* < 0.05) (Additional file 2: Fig. S7A-C).

Given that the disease stage, TIL, and 6-gene IFN-g gene signature have been shown to be highly predictive of the response to ICI therapy [9, 11, 69], we speculated that they might function as synergistic factors in predicting the response to immunotherapy. The genes of TILs (represented by CTLs) were extracted from Jiang et al., including CD8A, CD8B, GZMA, GZMB, and PRF1 [11]. The CTL score was estimated by the ssGSEA method. Therefore, a nomogram was developed to combine the mutation-based gene set with the disease stage, CTL, and 6-gene IFN-g gene signature to offer clinicians a quantitative approach for predicting OS in ICI-treated patients. The nomogram was constructed in the Riaz cohort (Fig. 2E), and the calibration curve of the nomogram showed good agreement between the observations and the predictions (Fig. 2F), suggesting that the mutation-based gene set, disease stage, CTL, and 6-gene IFN-g gene signature should be integrated into a predictive nomogram for ICI therapy.

### Underlying extrinsic immune landscapes of the high- and low-risk groups

**Fig. 3:**
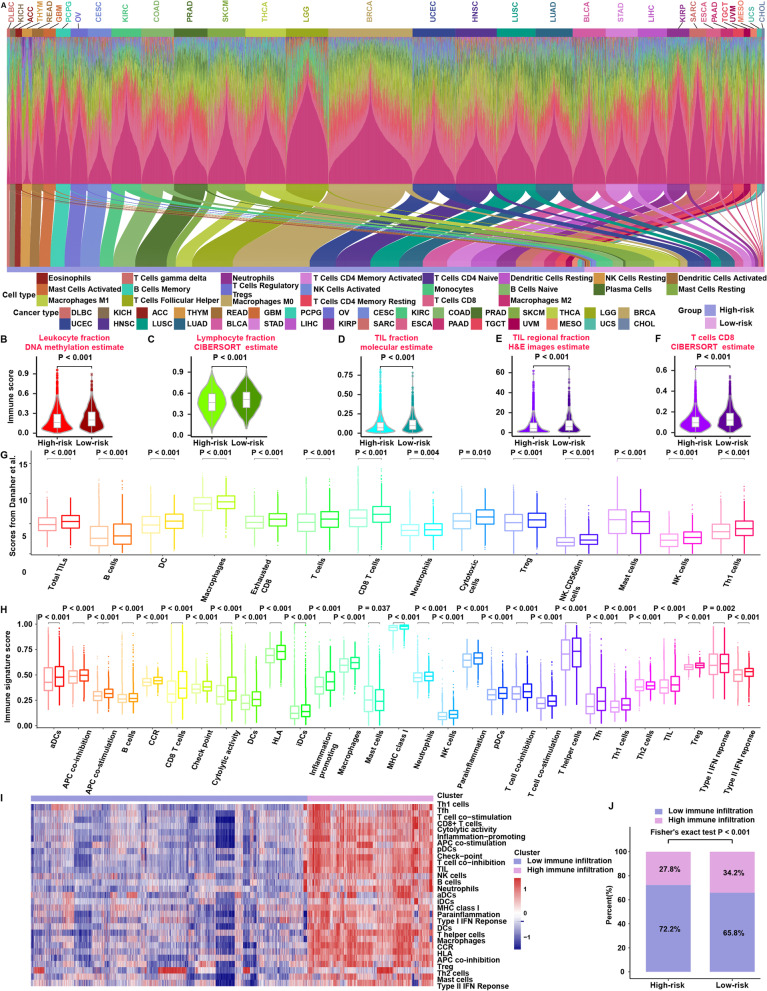
Immune landscapes of the high-risk and low-risk groups in the cohort from TCGA. **A** Bar charts depicting proportions of 22 types of immune cells estimated by the CIBERSORT method based on RNA-sequencing data for each patient and Sankey diagram showing that the patients in the cohort from TCGA were classified into high-risk and low-risk groups. **B** Comparison of leukocyte fractions based on DNA methylation data between the high-risk and low-risk groups. **C** Comparison of lymphocyte fractions estimated by the CIBERSORT method based on RNA-sequencing data between the high-risk and low-risk groups. **D** Comparison of TIL fractions based on molecular estimates from the processing of cancer genomics data between the high-risk and low-risk groups. **E** Comparison of TIL regional fractions based on estimates from processing diagnostic H&E images between the high-risk and low-risk groups. **F** Comparison of CD8 T cells estimated by the CIBERSORT method based on RNA-sequencing data between the high-risk and low-risk groups. **G** Comparison of 14 immune cells estimated by the Danaher method based on RNA-sequencing data between the high-risk and low-risk groups. In each cell type, the light color represents the high-risk group, and the dark color represents the low-risk group. The *P* value is shown at the top of the graph. **H** Comparison of the 29 immune signatures estimated by the ssGSEA method based on RNA-sequencing data between the high-risk and low-risk groups. For each cell type, the light color represents the high-risk group, and the dark color represents the low-risk group. The *P* value is shown at the top of the graph. **I** Unsupervised clustering based on 29 immune signatures in the cohort from TCGA, yielding two stable immune subtypes. **J** The proportions of high immune infiltration and low immune infiltration estimated by 29 immune signatures in the high-risk and low-risk groups

**Fig. 4:**
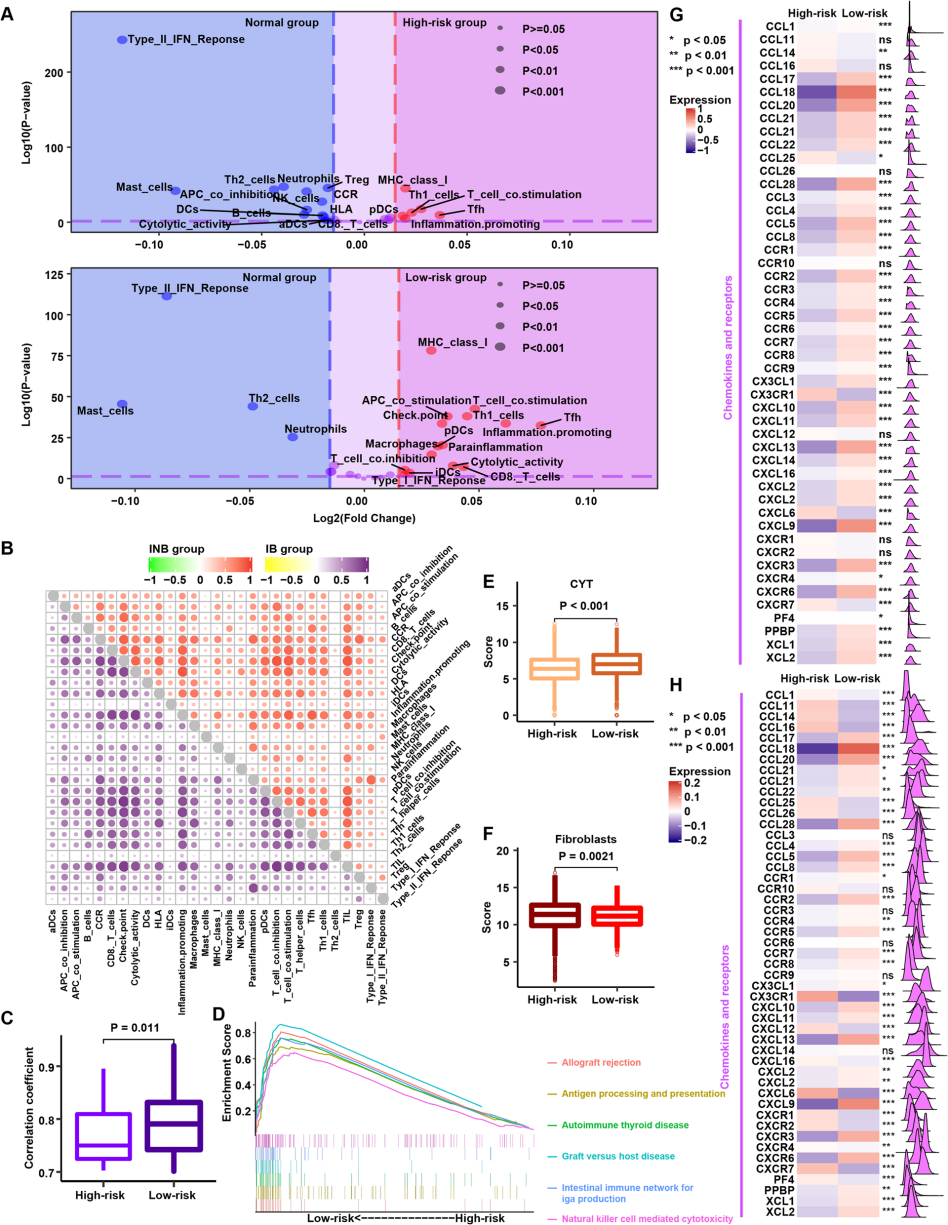
Potential extrinsic immune response landscapes in the high-risk and low-risk groups. **A** Volcano plots of 29 immune signatures in the high-risk and low-risk groups. Immune signatures enriched in cancer tissues are marked in red; immune signatures enriched in normal tissues are marked in blue. **B** Correlations among 29 immune signatures in the high-risk (top right panel) and low-risk (low left panel) groups. **C** Comparison of the correlation coefficient among 29 immune signatures between the high-risk and low-risk groups. Correlation coefficients greater than 0.7 were included in the analysis. **D** Gene set enrichment analysis of the high-risk and low-risk groups. **E** Comparison of the CYT score between the high-risk and low-risk groups. **F** Comparison of fibroblast abundances between the high-risk and low-risk groups. **G** Comparison of the expression patterns of chemokines between the high-risk and low-risk groups. **H** After normalizing the data according to the immune cell fraction, the expression patterns of chemokines were compared between the high-risk and low-risk groups

To further explore the relationship between the immune system and mutation-based gene sets, we performed multiomics analysis of the cohort from The Cancer Genome Atlas (TCGA). Using the same formula and cutoff obtained from the training cohort, the cohort from TCGA was classified into high-risk and low-risk groups (Fig. 3A). Comparison at the genomic level revealed larger leukocyte, lymphocyte, and TIL fractions in the low-risk group than in the high-risk group (*P* < 0.001) (Fig. 3B–D). In addition, we used the TIL fraction data according to Saltz et al., who applied deep learning methods to estimate TILs on hematoxylin and eosin-stained (H&E-stained) slides [45]. Strikingly consistent results for the H&E estimates of the TIL fraction were obtained (*P* < 0.001) (Fig. 3E). In detail, the proportion of immune-stimulatory cells (such as CD8 T cells) was significantly increased in the low-risk group compared with the high-risk group (*P* < 0.001) (Fig. 3F). To further examine the above results using different methods of evaluating immune cells, we analyzed their distribution between the high- and low-risk groups according to the immune infiltration scores from Danaher et al. (Fig. 3G) and immune signature scores (Fig. 3H). The low-risk group was characterized by a greater abundance of immune cells, such as TILs and CD8 T cells (*P* < 0.05) (Fig. 3G, H). TCGA cohort patients were then clustered on the basis of immune signature scores using unsupervised clustering to assess whether the high-risk and low-risk groups correctly corresponded to the low-immune infiltration and high-immune infiltration groups, and unsupervised clustering revealed two distinct immune patterns with high and low levels of immune infiltration (Fig. 3I). Interestingly, the high immune infiltration group was significantly enriched in cases from the low-risk group (Fig. 3J). In addition, in the low-risk group, the immune signature scores at the tumor site were obviously greater than those at the normal site; conversely, the immune signature scores of the high-risk group at the tumor site were obviously lower than those at the normal site (Fig. 4A). Furthermore, the correlation among immune activities in the low-risk group was significantly higher than that in the high-risk group (Fig. 4B, C). GSEA showed significant enrichment in 13 pathways in the low-risk group, including 6 immune-related pathways, such as “natural killer cell mediated cytotoxicity” (*P* < 0.05) (Additional file 1: Table S6) (Fig. 4D). In contrast, no enrichment in any immune-related pathway was observed for the high-risk group (Additional file 1: Table S7). Low-risk tumors were associated with significantly higher CYT scores (*P* < 0.001) (Fig. 4E), and a significantly larger number of fibroblasts was found in the high-risk group (*P* < 0.01) (Fig. 4F). According to these results, the low-risk group showed abundant immune cells at the tumor site, which led to a response to ICI therapy, whereas fibroblasts may contribute to extrinsic immune escape in the high-risk group.

Furthermore, we found higher expression of chemokines in the low-risk group (Fig. 4G), which was compatible with the higher infiltration of immune cells in this group (Fig. 3). To provide a fair comparison normalized to immune cell density in tissues, we divided the expression of these genes by the immune cell fraction and compared them again (Fig. 4H); the results obtained after normalization were generally consistent with those obtained before normalization. Therefore, we infer that enrichment of chemokines may invoke an immune response in the low-risk group.

### Underlying intrinsic immune landscapes of the high- and low-risk groups

**Fig. 5:**
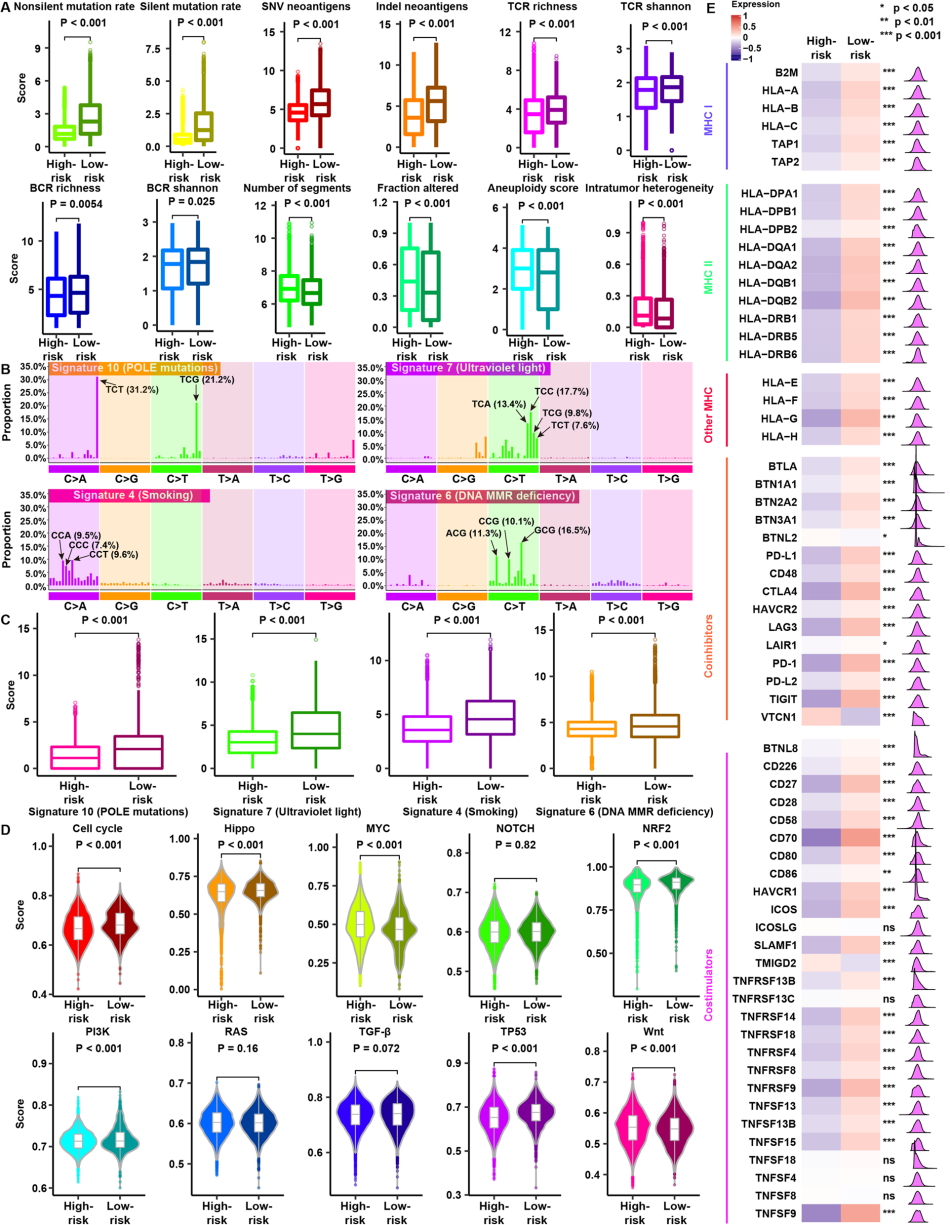
Potential intrinsic immune response and escape landscapes in the high-risk and low-risk groups. **A** Comparison of immunogenomic indicators between the high-risk and low-risk groups. **B** Mutational activities of four corresponding extracted mutational signatures. **C** Comparison of four mutational signatures between the high-risk and low-risk groups. **D** Comparison of enrichment scores of 10 oncogenic pathways between the high-risk and low-risk groups. **E** Comparison of the expression patterns of MHC molecules, costimulators and coinhibitors between the high-risk and low-risk groups

We first compared some underlying factors determining tumor immunogenicity between the two groups. The low-risk group showed a higher mutation rate and neoantigen load than the high-risk group (all *P* < 0.001) (Fig. 5A), as well as significantly higher TCR diversity and BCR diversity (*P* < 0.001) (Fig. 5A). Compared with the low-risk group, the high-risk group exhibited a higher CNV burden and aneuploidy (all *P* < 0.001) (Fig. 5A). This result is consistent with the previous discovery that tumor aneuploidy is related to a reduced response to immunotherapy and to markers of immune evasion [70]. In terms of intertumoral heterogeneity, patients in the high-risk group displayed higher intertumoral heterogeneity than those in the low-risk group (*P* < 0.001) (Fig. 5A). This result further supports the concept that in the presence of cytolytic activity and fewer actively infiltrating immune cells, the tumor is allowed to clonally evolve, promoting the development of heterogeneity. Hence, we conclude that high immunogenicity may cause an extrinsic immune response in the low-risk group.

To further understand the mutational processes in the high-risk and low-risk groups, we delineated the mutational signatures based on somatic mutation data and identified four distinct patterns of mutagenesis in the cohort from TCGA (Fig. 5B). Signature 10 (5.58%), which contains a predominance of C>A mutations at TCT (31.2%) sites and C>T mutations at TCG (21.2%) sites, has been previously related to altered activity of the error-prone polymerase POL ε (POLE) as a consequence of mutations in the gene (Fig. 5B). Signature 7 (24.75%) contains an extremely strong transcriptional strand bias for C>T mutations in the CpTpN context, possibly due to ultraviolet light exposure (Fig. 5B). Signature 4 accounts for 31.63% of all point mutations and is characterized by C>A mutations; it may be associated with smoking (Fig. 5B). Signature 6 (38.03%), which is the most prevalent signature, is characterized by C>T mutations and thought to be associated with defective DNA mismatch repair (MMR); this signature has been detected in microsatellite unstable tumors (Fig. 5B). The four signatures were found at obviously higher frequencies in the low-risk group than in the high-risk group (all *P* < 0.001) (Fig. 5C). Smoking signatures and MMR signatures have been reported to be associated with immune response [14, 71]. We then calculated enrichment scores for oncogenes in 10 common oncogenic pathways in the low- and high-risk groups [50]. The cell cycle, Hippo, NRF2, PI3K, and TP53 pathways had higher scores in the low-risk group, whereas the MYC and Wnt pathways were enriched in the high-risk group (all *P* < 0.001) (Fig. 5D). The Wnt pathway has been shown to be related to immune exclusion [72].

Compared to the low-risk group, the high-risk group expressed smaller amounts of MHC I- and II-related antigen-presenting molecules (all *P* < 0.001), resulting in intrinsic immune escape (Fig. 5E). In contrast, the low-risk group had higher expression of most MHC genes, which is indicative of stronger immunogenicity. We also found immune checkpoint molecules (such as PD-1, PD-L1, and CTLA4) and costimulatory molecules to be more highly expressed in the low-risk group than in the high-risk group (most *P* < 0.001) (Fig. 5E). Therefore, we conclude that these immune checkpoint molecules cause a response to ICI therapy.

The above research was based on the mutation-based gene set as a whole to study the potential mechanisms of immune response and escape; thus, we further characterized the presumed mechanism by which each gene is related to the response to immunotherapy. We first compared the nonsilent mutation rate between the mutant and wild-type status of each gene in the cohort from TCGA. The nonsilent mutation rate was significantly higher in tumors with mutant genes than in those with wild-type genes (Additional file 2: Fig. S8A), indicating that mutations are related to enhanced tumor immunogenicity.

In addition, based on molecular estimates, TILs were more abundant in mutant-gene tumors than in wild-type-gene tumors (Additional file 2: Fig. S8B), which was validated using the H&E estimate data (Additional file 2: Fig. S8C). Next, we focused on T cells and found significantly higher TCR richness in tumors harboring mutant genes than in tumors with wild-type genes (Additional file 2: Fig. S9A). Based on CIBERSORT data, CD8 T cells were more abundant in mutant-gene tumors than in wild-type-gene tumors (Additional file 2: Fig. S9B), and these results were validated using immune signature score (Additional file 2: Fig. S9C). To better characterize the immune profile, differences in the expression pattern of immune checkpoint genes between mutant- and wild-type-gene tumors were explored. In line with the data for TILs, PD-1, PD-L1, and CTLA4 were upregulated in tumors with mutant genes (Additional file 2: Fig. S10). These results suggest that mutation of these 11 genes is strongly related to a hot immune microenvironment and enhanced tumor immunogenicity, which firmly supports the predictive abilities of these mutations for ICI therapy.

We also compared the lymphocyte, immune activation and mutation signature 7 (ultraviolet radiation, UVR) between the low-risk group and the high-risk group in the breast invasive carcinoma (BRCA) and skin cutaneous melanoma (SKCM) cohorts (Additional file 2: Fig. S7D-I); lymphocyte genes (represented by CTLs) were extracted from Jiang et al., including CD8A, CD8B, GZMA, GZMB, and PRF1 [11], and immune activation genes (represented by T and NK cell activity markers) were extracted from Wan et al., including GZMA, GZMB, IFNG, and NKG7 [50]. The lymphocyte score and immune activation score for each sample were estimated by the ssGSEA method. Compared with the high-risk group, the low-risk group showed a higher lymphocyte count, stronger immune activation and a higher mutation signature 7 (UVR) score in both cohorts (all *P* < 0.05, Additional file 2: Fig. S7D-I).

Furthermore, to balance the bias of the number of high- and low-risk groups among different cancer types, we selected 50 high-risk cases and 50 low-risk cases from each cancer type that had at least these numbers of cases, including bladder urothelial carcinoma (BLCA), BRCA, cervical squamous cell carcinoma and endocervical adenocarcinoma (CESC), colon adenocarcinoma (COAD), head and neck squamous cell carcinoma (HNSC), kidney renal clear cell carcinoma (KIRC), lung adenocarcinoma (LUAD), lung squamous cell carcinoma (LUSC), SKCM, stomach adenocarcinoma (STAD), thyroid carcinoma (THCA), and uterine corpus endometrial carcinoma (UCEC) (Additional file 2: Fig. S7J). We conducted 1000 random samplings and compared the lymphocyte score, immune activation score and mutation signature 7 (UVR) between the low-risk and high-risk groups and found higher scores in the low-risk group (all *P* < 0.001, Additional file 2: Fig. S7K-M).

### Copy number features of the high- and low-risk groups

**Fig. 6:**
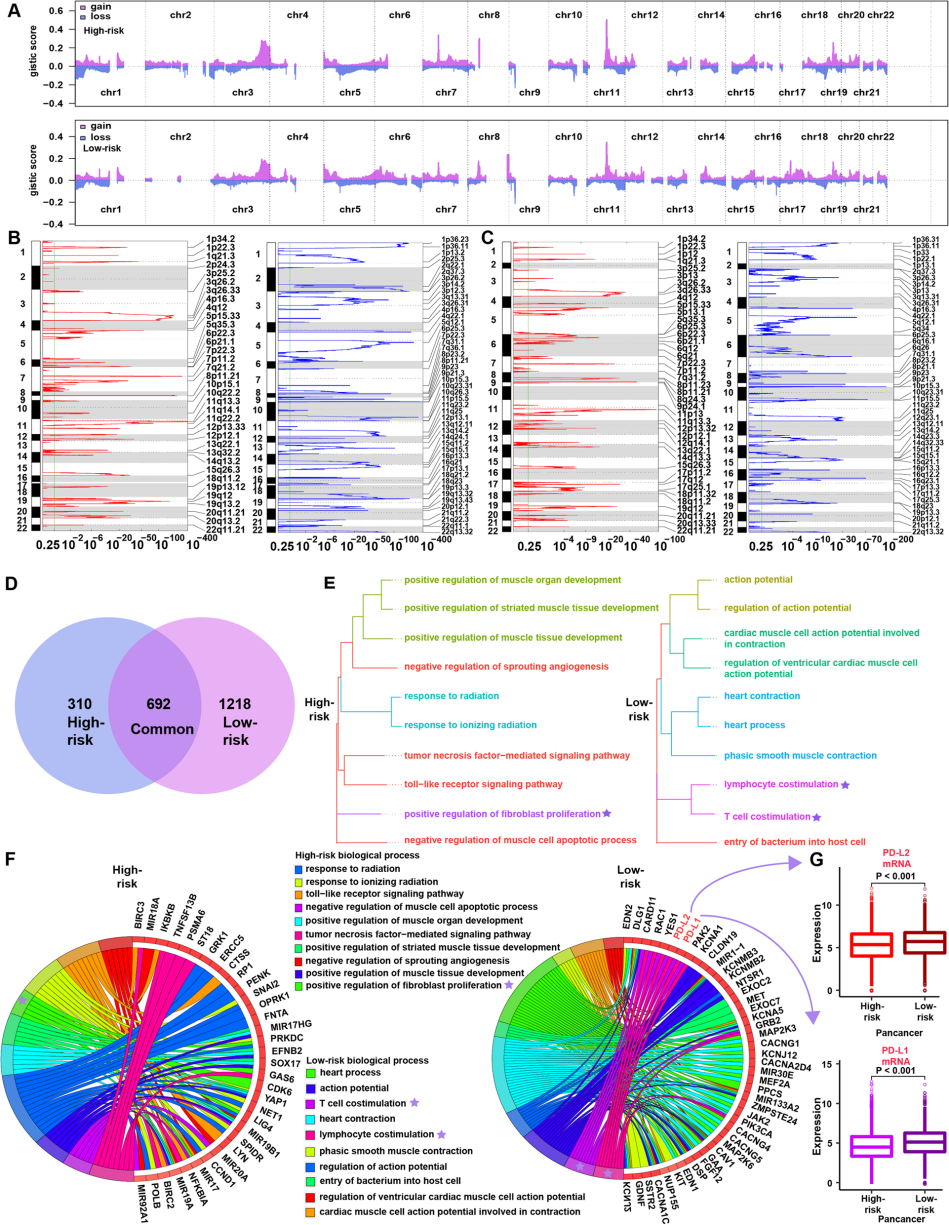
Copy number alterations in the high-risk and low-risk groups. **A** Copy number profiles of the high-risk (above) and low-risk (below) groups, with gains shown in red and losses shown in blue. **B** Detailed cytobands with focal amplification (left) and deletion (right) peaks identified in the high-risk group. **C** Detailed cytobands with focal amplification (left) and deletion (right) peaks identified in the low-risk group. **D** Venn diagrams showing significantly amplified genes in the high-risk and low-risk groups. Each circle in the Venn diagram represents one group, and the number in the overlaid area represents common genes between the groups. **E** Cluster analysis of the top 10 biological processes in the high-risk (left) and low-risk (right) groups. **F** Circular plot of the top 10 biological processes and corresponding enriched genes in the high-risk (left) and low-risk (right) groups. **G** Comparison of the mRNA expression of PD-L1 and PD-L1 between the high-risk and low-risk groups in the cohort from TCGA

Significant differences in chromosomal aberrations were detected between the high-risk and low-risk groups (Fig. 6A). Compared with the high-risk group (Fig. 6B), focal amplification peaks were observed for well-characterized immune genes, such as PD-L1 (9p24.1) and PD-L2 (9p24.1), in the low-risk group (Fig. 6C). Venn diagrams revealed 692 shared genes in the chromosome regions with copy number amplification in both groups, with 310 and 1218 genes specifically amplified in the high-risk and low-risk groups, respectively (Fig. 6D). We annotated these specific amplified genes through biological processes in Gene Ontology (Additional file 1: Tables S8 and S9) and then clustered the top 10 biological processes (Fig. 6E). The low-risk group was significantly enriched in 2 immune-related biological processes, “lymphocyte costimulation” and “T cell costimulation” (Fig. 6E). In contrast, the high-risk group was significantly enriched in “positive regulation of fibroblast proliferation” but not any immune-related biological process (Fig. 6E). This result was surprisingly consistent with previous results; that is, there were more immune cells in the low-risk group (Fig. 3B–D) and more fibroblasts in the high-risk group (Fig. 4F). Notably, PD-L1 and PD-L2 (located in the low-risk group-specific amplification peak 9p24.1) were annotated in both immune-related biological processes, indicating that PD-L1 and PD-L2 may play important roles in regulating immune status in the low-risk group (Fig. 6F). At the level of mRNA expression in the cohort from TCGA, we found significantly higher mRNA expression of PD-L1 and PD-L2 in the low-risk group (Fig. 6G), consistent with the CNV data. This finding indicates that CNVs in tumors contribute to observed differences in immune infiltration.

## Discussion

Predictive biomarkers may help members of the medical community offer accurate guidance for ICI-treated patients, aid in cost management, and accelerate clinical trials and FDA approvals. Several biomarkers have been investigated, and some have been used to predict treatment outcomes. Indeed, recent studies have shown a robust association between TMB and the response to ICIs [73]. However, some patients with a high TMB may carry decisive mutations (in B2M, JAK1/2, etc.) that are closely associated with immunotherapy resistance, leading to a lack of response to ICIs and indicating that the TMB is insufficient for prognosis prediction [17]. Therefore, it is necessary to identify alternative markers of responsiveness. Based on a cohort of 2504 patients with different types of cancer, we established and validated a mutation-based gene set including 11 genes to predict survival benefits in patients undergoing ICI therapy. To the best of our knowledge, the current study is the first to investigate a comprehensive mutation-based gene set across different tumor types using independent cohorts.

Different types of tumors were included in our study, and different types of tumors have different prognoses. Therefore, we focused on eliminating bias among different types of tumors in the establishment and evaluation of the mutation-based gene set. First, we used the PSM adjustment method to adjust for bias among different types of tumors. The PSM algorithm is an important statistical tool to control confounding in observational studies, and it has been widely used in clinical research and pancancer genomic studies to reweight potential confounding effects in a multivariate manner [33, 34, 74–77]. In addition, the performance of methods that correct the confounder effect by balancing the propensity score was reported to be superior to that of other methods, including the t test, analysis of variance (ANOVA) and general linear model (GLM) [33]. Therefore, to identify gene mutations associated with prognosis, we employed a propensity score algorithm to reduce potential confounding effects among different types of tumors. Second, after the mutation-based gene set was established, we investigated whether the mutation-based gene set was restricted to specific groups or applicable to different populations. Stratification analyses indicated that the mutation-based gene set was significantly associated with OS in patients treated with ICI therapy, regardless of whether the cancer type was advanced lung cancer or colorectal cancer. The results of the subgroup analysis were consistent with the results of PSM adjustment. Third, we performed multivariate Cox regression analysis and found that our mutation-based gene set was independent of tumor type in predicting prognosis. In summary, we tested the application performance of the mutation-based gene set across different types of tumors using the PSM algorithm, stratified analysis, and multivariate Cox regression analysis, and based on the results, we believe the mutation-based gene set to be reliable.

Furthermore, we employed the multidimensional TCGA dataset to analyze how cancers respond to immunotherapy. We found that the low-risk group featured an inflammatory pattern of immune activities, such as high levels of CD8 T cell infiltration determined by the ESTIMATE approach, and stronger immunogenicity, such as a higher TMB. When we utilized the immune infiltration scores from Danaher et al. and the ssGSEA approach to calculate overall immune cell infiltration levels for cancers, the immune score was significantly higher in the low-risk group than in the high-risk group, which again confirmed the stronger antitumor immune activity in the former group. Many studies have shown that the density of TILs is positively associated with the immune response in patients with various kinds of cancers [78]. In addition to a high level of cytotoxic T cell infiltration, the low-risk group was characterized by overexpression of immune checkpoints, such as PD-L1, PD-1, and CTLA-4, compared with the high-risk group. Therefore, activated antitumor immunity, high PD-L1, PD-L1, and CTLA-4 expression, and enhanced t1umor immunogenicity might explain why the low-risk group was found to be more likely than the high-risk group to benefit from ICI therapy.

Our research has the following innovations and practical application. First, our study investigated different types of tumors (such as NSCLC, melanoma, and renal cell carcinoma), which represent the most common types of cancers treated with ICI therapy [79–81]. Several mRNA-based signatures, such as the T cell-inflamed gene-expression profile (GEP), an 18-gene assay, have been developed to predict clinical efficacy in patients undergoing ICI therapy [82]. To the best of our knowledge, the current study is the first to investigate a comprehensive mutation-based gene set across different tumor types using independent cohorts. Second, the application of multibiomarker predictive models requires an understanding of the factors that influence the accuracy and precision of high-throughput-based assays in clinical practice. Principal among these factors is the variability of biomarker measurements, which can be classified into preanalytical (intrinsic to the sample) and technical (intrinsic to the platform) sources of variation. Tissue-specific variability influences mRNA expression and is controlled by introducing several reference genes; relative quantitation is adopted to assess mRNA expression by normalization to reference genes. The risk score formulas and threshold values of these mRNA signatures are not suitable for validation using other types of measurement data. In the current study, we developed a mutation-based gene set to predict the clinical efficacy of ICI therapy. The composition of the above mutations is neither affected by the tissue type nor adjusted for by any other biomarker. However, the risk score formula as well as the threshold value for the mutation-based gene set can be validated by other tumor analysis methods, such as DNA sequencing and single-nucleotide polymorphism microarray analysis. Hence, the mutation-based gene set is not affected by technical sources of variation, even when using different platforms for different centers. Third, in practice, the mutation-based gene set avoids exposing patients to potential immune-related adverse effects if they are unlikely to respond and enables matching of a patient to a potentially more effective treatment sooner. In addition, given that the treatment course typically costs more than $120 000 on average [73], the application of biomarker strategies that improve diagnostic accuracy may help avoid considerable costs for what is anticipated to be a substantially reduced benefit. Overall, a mutation-based gene set incorporating these alterations should be assessed due to the greater ease of obtaining tumor specimens from patients on the basis of targeted NGS of these genes rather than assessing the TMB, which is complicated and expensive in routine practice. Fourth, we compared prediction performance between the mutation-based gene set and other factors that can predict immunotherapy, including the frameshift indel mutation burden, tobacco mutation signature, UV signature, APOBEC signature, DNA damage response pathway mutations, B2M mutation, JAK1 mutation, JAK2 mutation, KRAS mutation, TP53 mutation, PTEN mutation, STK11 mutation, and BAP1 mutation. We found that the prediction performance of the mutation-based gene set was superior to that of all of those factors.

Several limitations of this study should be considered. First, as some mutations may be enriched in some tumor types, the original goal of this study was to create a panel rather than identify a single gene (such as BRAF), as the former can include more genes to predict prognosis across different types of tumors. In addition, we explored all pancancer articles and evaluated how other researchers eliminated the biases associated with different types of tumors. Because we found that the PSM adjustment method is well recognized [33, 34, 74–77], we used PSM adjustment in this study to eliminate such bias. We also included different types of tumors as much as possible to eliminate these biases. To the best of our knowledge, this study is the largest to date to explore prognosis prediction for mutation-based pancancer immunotherapy. Of course, as large sample sizes of immunotherapy cohort clinical trials and better algorithms continue to be published, we will update our mutation-based gene set accordingly in the future to make it more comprehensive. Second, although we explored the immune landscape of each of the 11 genes in the mutation-based gene set, we still need to elucidate the molecular mechanism underlying the influence of each gene on immunotherapy in in vivo and in vitro functional experiments. Third, the enrichment scores of oncogenic pathways and expression patterns of immune checkpoints should also be examined by immunohistochemistry.

## Conclusions

To use high-throughput methodologies in clinical practice, a marker must be validated by utilizing widely available tissues, such as formalin-fixed and paraffin-embedded tumor tissues. Once this major step has been achieved, we will enter a new era of truly tailored and precision medicine, likely with higher cure rates. Our mutation-based gene set meets the above requirement and is the first systematically identified comprehensive genomic marker for assessing the effect of ICI therapy across a broad spectrum of cancers. This study also represents the largest prognostic model discovery project for cancer patients who received ICI treatment (either as monotherapy or as a combination of anti-PD-1 and anti-CTLA-4). The nomogram combining the mutation-based gene set with the TMB and drug type can help clinicians select patients who have a strong likelihood of responding to ICI therapies. In addition, our study revealed distinct immune landscapes for the high- and low-risk groups. Specific genomic alterations might drive the formation of these microenvironment phenotypes. Overall, this work proposes a new tumor classification system with the potential to guide ICI treatment decisions.

## Supplementary Information

**Additional file 1: Table S1.** The clinical data for each sample used in the training cohort. **Table S2.** The clinical data for each sample used in the validation cohort. **Table S3.** The clinical data for each sample used in the TCGA cohort. **Table S4.** Twenty-nine immune signatures were used to assess immune status. **Table S5.** Frequency of nonzero coefficients for 98 prognostic genes in 1000 LASSO analyses. **Table S6.** Gene sets enriched in the low-risk group. **Table S7.** Gene sets enriched in the high-risk group. **Table S8.** Functional enrichment analysis of 310 genes specifically amplified in the high-risk group. **Table S9.** Functional enrichment analysis of 1,218 genes specifically amplified in the low-risk group.

**Additional file 2: Fig. S1.** Flowchart of the clinical cohort consolidation. **Fig. S2.** Flowchart of the construction and validation of the mutation-based gene set and the summary of immune landscapes in the high-risk and low-risk groups. **Fig. S3.** Assessment of the predictive performance of the mutation-based gene set. **Fig. S4.** Subgroup analysis of the mutation-based gene set in the training cohort. **Fig. S5.** Comparison of C-indexes for the mutation-based gene set with other predictors. **Fig. S6.** The distribution of the low-risk group and the high-risk group for each cancer type in each dataset. **Fig. S7.** Comparison of immune activity between the low-risk group and the high-risk group. **Fig. S8.** Mutations in 11 genes in the mutation-based gene set are related to enhanced tumor immunogenicity and high immune cell infiltration. **Fig. S9.** Mutations of 11 genes in the mutation-based gene set are related to high T cell infiltration. **Fig. S10.** Mutations of 11 genes in the mutation-based gene set are associated with high immune checkpoint expression.

## Data Availability

All data used in this study are from public datasets and can be accessed without restriction. The web links or unique identifiers for public datasets are described in the paper. The codes used for data analysis in this manuscript have been deposited in GitHub (https://github.com/longjunyu/Pancan-ICI) [83].

## Abbreviations

ACC: Adrenocortical carcinoma
aDCs: Activated dendritic cells
APCs: Antigen-presenting cells
AUC: Area under the curve
BCR: B cell receptor
BLCA: Bladder urothelial carcinoma
BRCA: Breast invasive carcinoma
CCR: Cytokine and cytokine receptor
CESC: Cervical squamous cell carcinoma and endocervical adenocarcinoma
CHOL: Cholangiocarcinoma
CNV: Copy number variation
COAD: Colon adenocarcinoma
COSMIC: Catalogue of somatic mutations in cancer
CTLA-4: Cytotoxic T lymphocyte-associated protein-4
CYT: Cytolytic activity score
DCs: Dendritic cells
DLBC: Lymphoid neoplasm diffuse large B cell lymphoma
ES: Enrichment score
ESCA: Esophageal carcinoma
ESTIMATE: Estimation of stromal and immune cells in malignant tumors using expression data
FDR q-val: False discovery rate *q* value
FWER P-val: Family-wise-error rate *P* value
GBM: Glioblastoma multiforme
GEP: Gene expression profile
GO: Gene Ontology
GSEA: Gene set enrichment analysis
HLA: Human leukocyte antigen
HNSC: Head and neck squamous cell carcinoma
ICI: Immune-checkpoint inhibitor
iDCs: Immature dendritic cells
IDI: Integrated discrimination improvement
IFN: Interferon
Indel: Insertion-deletion
ITH: Intertumoral heterogeneity
KEGG: Kyoto Encyclopedia of Genes and Genomes
KICH: Kidney chromophobe
KIRC: Kidney renal clear cell carcinoma
KIRP: Kidney renal papillary cell carcinoma
LAML: Acute myeloid leukemia
LASSO: Least absolute shrinkage and selection operator
LGG: Low-grade glioma
LIHC: Liver hepatocellular carcinoma
LUAD: Lung adenocarcinoma
LUSC: Lung squamous cell carcinoma
MESO: Mesothelioma
MHC: Major histocompatibility complex
MMR: Mismatch repair
MSI: Microsatellite instability
NES: Normalized enrichment score
NGS: Next-generation sequencing
NK: Natural killer
NMF: Nonnegative matrix factorization
NOM p-val: Nominal *P* value
NRI: Net reclassification improvement
NSCLC: Non-small-cell lung cancer
OS: Overall survival
OV: Ovarian serous cystadenocarcinoma
PAAD: Pancreatic adenocarcinoma
PCPG: Pheochromocytoma and paraganglioma
PD-1: Programmed death-1
pDCs: Plasmacytoid dendritic cells
PD-L1: Programmed death ligand-1
PRAD: Prostate adenocarcinoma
READ: Rectum adenocarcinoma
ROC: Receiver operating characteristic
SARC: Sarcoma
SCNA: Somatic copy number alteration
SKCM: Skin cutaneous melanoma
SNV: Single-nucleotide variant
ssGSEA: Single-sample gene set enrichment analysis
STAD: Stomach adenocarcinoma
TCGA: The Cancer Genome Atlas
TCR: T cell receptor
Tfh cells: Follicular helper T cells
TGCT: Testicular germ cell tumors
Th17 cells: T helper 17 cells
THCA: Thyroid carcinoma
THYM: Thymoma
TILs: Tumor-infiltrating lymphocytes
TMB: Tumor mutation burden
TME: Tumor microenvironment
Tregs: Regulatory T cells
UCEC: Uterine corpus endometrial carcinoma
UCS: Uterine carcinosarcoma
UVM: Uveal melanoma
