# Supplementary material for: A mutation-based gene set predicts survival benefit after immunotherapy across multiple cancers and reveals the immune response landscape
Source: Genome Med. 2022 Feb 24;14:20. doi: 10.1186/s13073-022-01024-y (PMC8867854; doi:10.1186/s13073-022-01024-y)
Supplement: Supplementary file 2 — Additional file 2: Fig. S1. Flowchart of the clinical cohort consolidation. Fig. S2. Flowchart of the construction and validation of the mutation-based gene set and the summary of immune landscapes in the high-risk and low-risk groups. Fig. S3. Assessment of the predictive performance of the mutation-based gene set. Fig. S4. Subgroup analysis of the mutation-based gene set in the training cohort. Fig. S5. Comparison of C-indexes for the mutation-based gene set with other predictors. Fig. S6. The distribution of the low-risk group and the high-risk group for each cancer type in each dataset. Fig. S7. Comparison of immune activity between the low-risk group and the high-risk group. Fig. S8. Mutations in 11 genes in the mutation-based gene set are related to enhanced tumor immunogenicity and high immune cell infiltration. Fig. S9. Mutations of 11 genes in the mutation-based gene set are related to high T cell infiltration. Fig. S10. Mutations of 11 genes in the mutation-based gene set are associated with high immune checkpoint expression. [file 13073_2022_1024_MOESM2_ESM.docx]

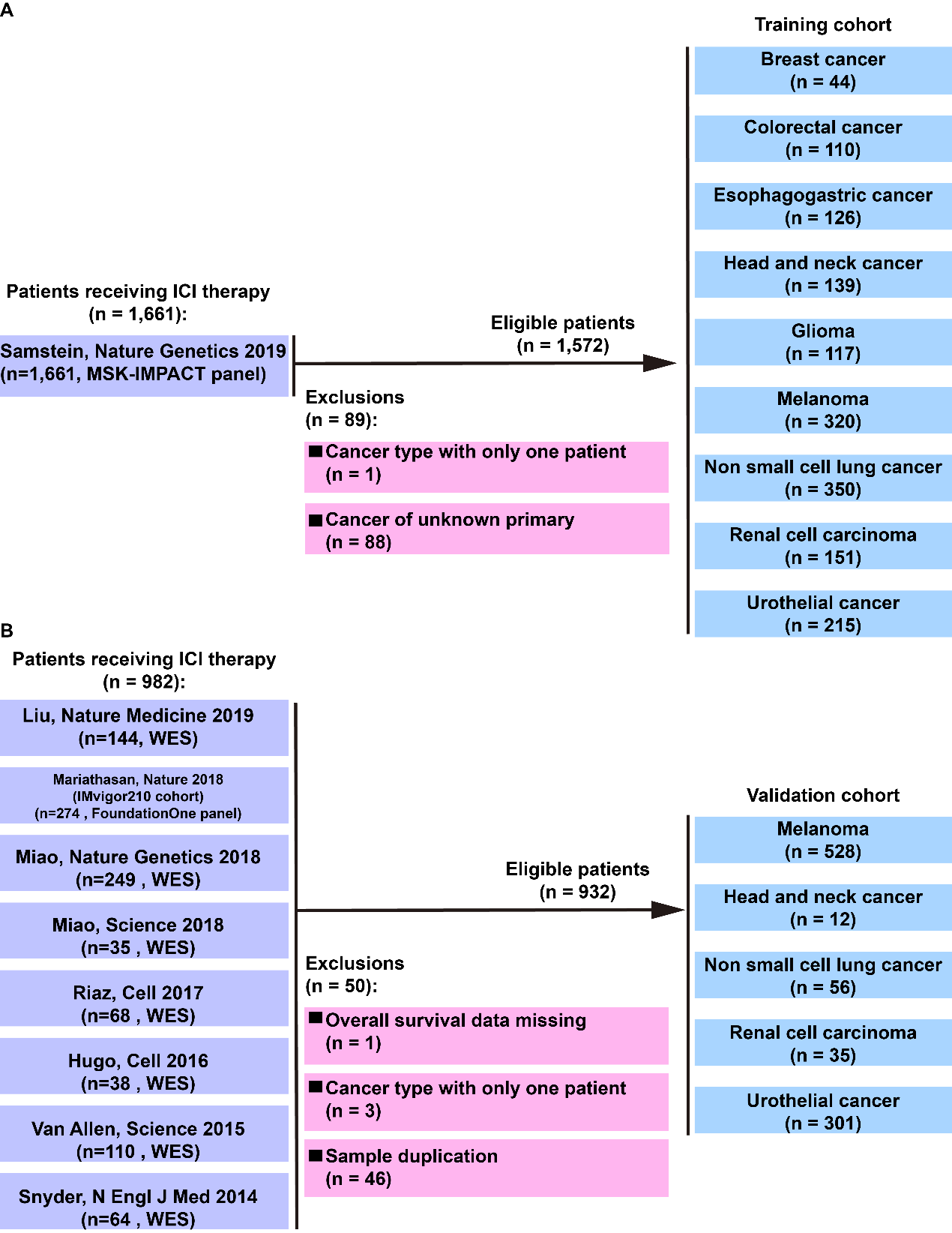


**Fig. S1: Flowchart of the clinical cohort consolidation.**


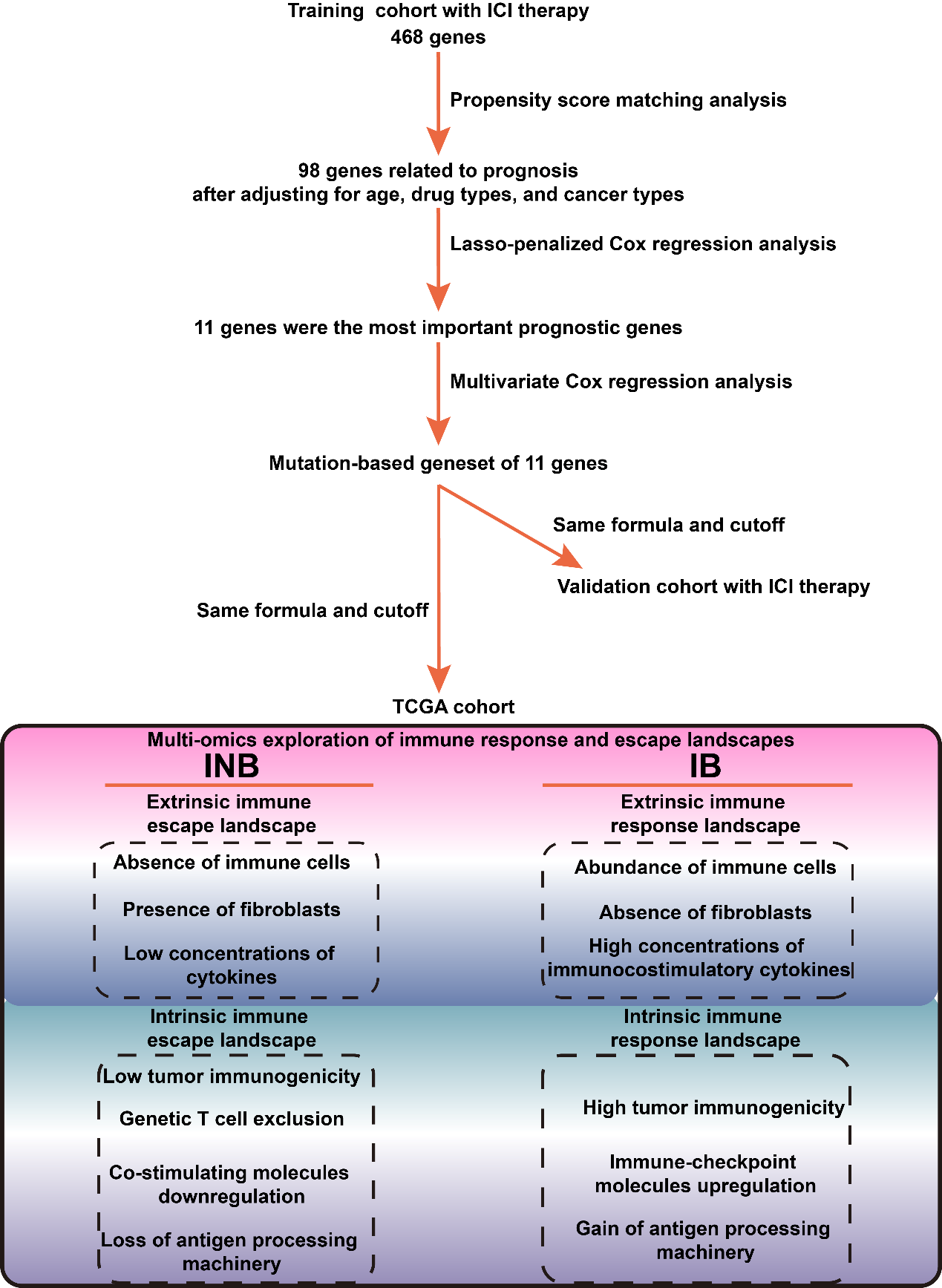


**Fig. S2: Flowchart of the construction and validation of the mutation-based gene set and the summary of immune landscapes in the high-risk and low-risk groups.**


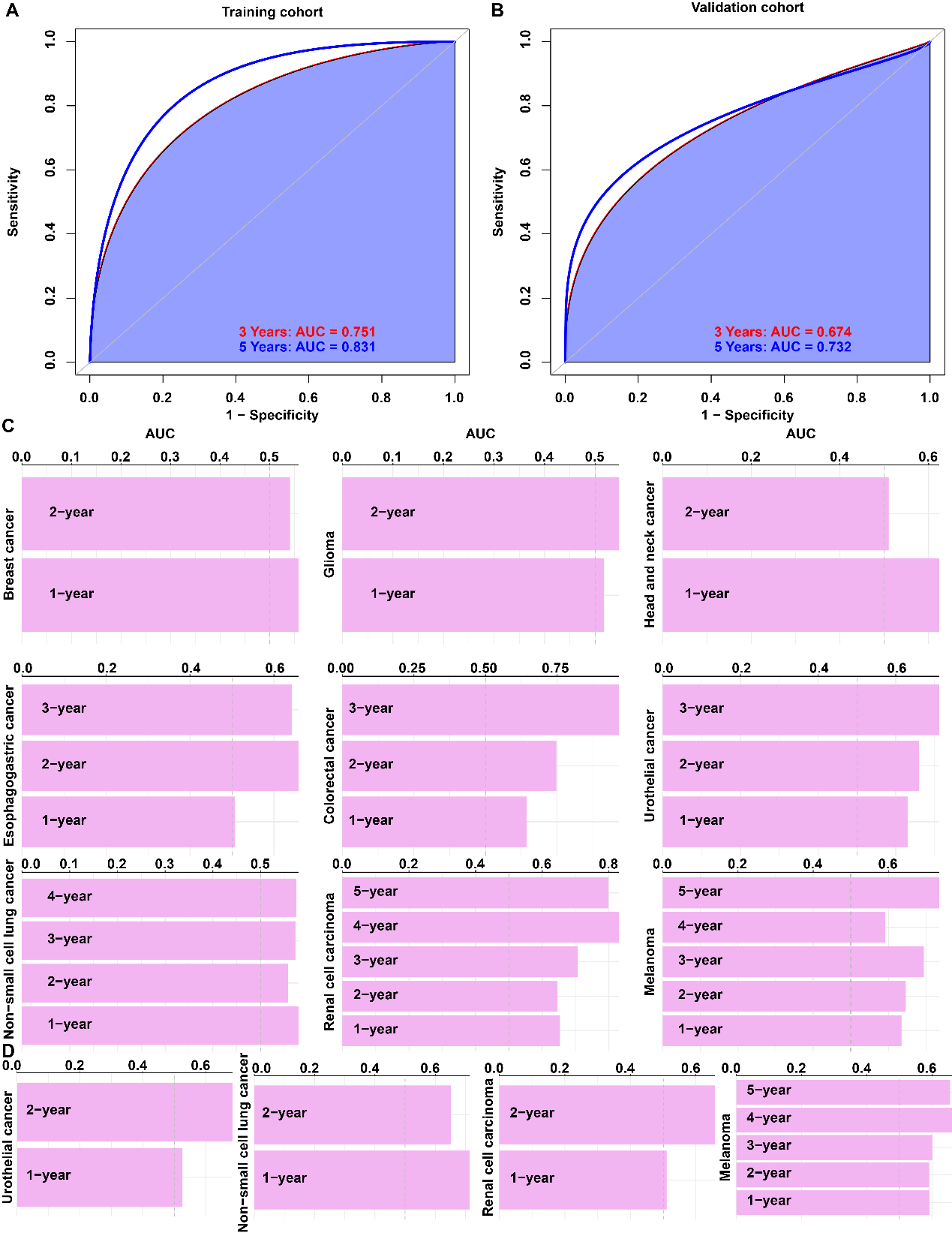


**Fig. S3: Assessment of the predictive performance of the mutation-based gene set.**

(A) Receiver operating characteristic (ROC) curve analysis of the training cohort. (B) ROC curve analysis of the validation cohort. (C) Area under the ROC curve (AUC) analysis for each cancer type in the training cohort. (D) AUC analysis for each cancer type in the validation cohort.


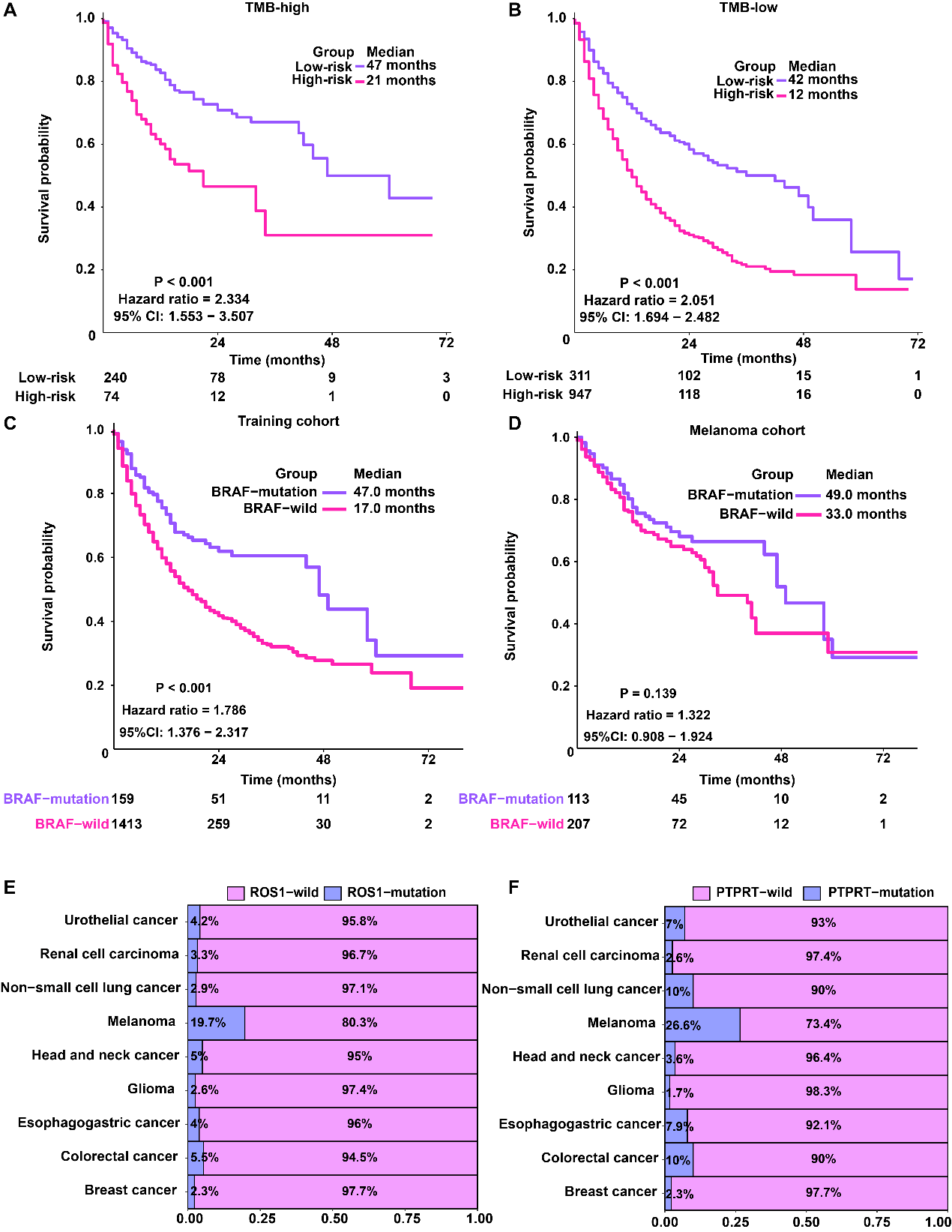


**Fig. S4: Subgroup analysis of the mutation-based gene set in the training cohort.**

(A) Survival analysis of the mutation-based gene set in the TMB-high group. (B) Survival analysis of the mutation-based gene set in the TMB-low group. (C) Survival analysis of the BRAF mutation status in the training cohort. (D) Survival analysis of the BRAF mutation status in the melanoma cohort. (E) The mutation frequency distribution of ROS1 in each cancer type. (F) The mutation frequency distribution of PTPRT in each cancer type.


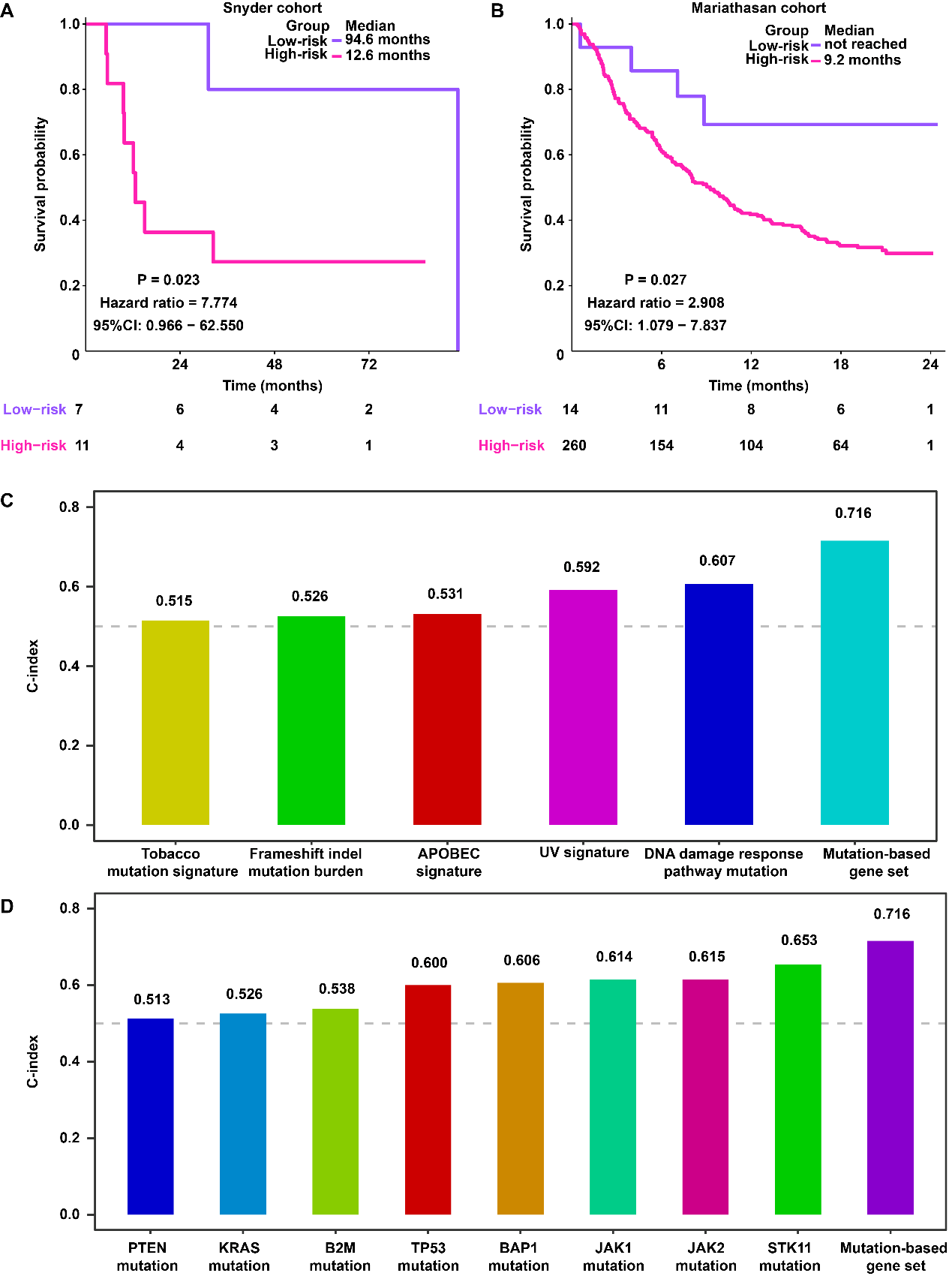


**Fig. S5: Comparison of C-indexes for the mutation-based gene set with other predictors.**

(A) Survival analysis of the mutation-based gene set in the Snyder cohort. (B) Survival analysis of the mutation-based gene set in the Mariathasan cohort. (C) Comparison of C-indexes between the mutation-based gene set and the frameshift insertion/deletion (indel) mutation burden, tobacco mutation signature, UV signature, APOBEC signature, and DNA damage response pathway mutation. (D) Comparison of C-indexes between the mutation-based gene set and B2M mutation, JAK1 mutation, JAK2 mutation, KRAS mutation, TP53 mutation, PTEN mutation, STK11 mutation, and BAP1 mutation.


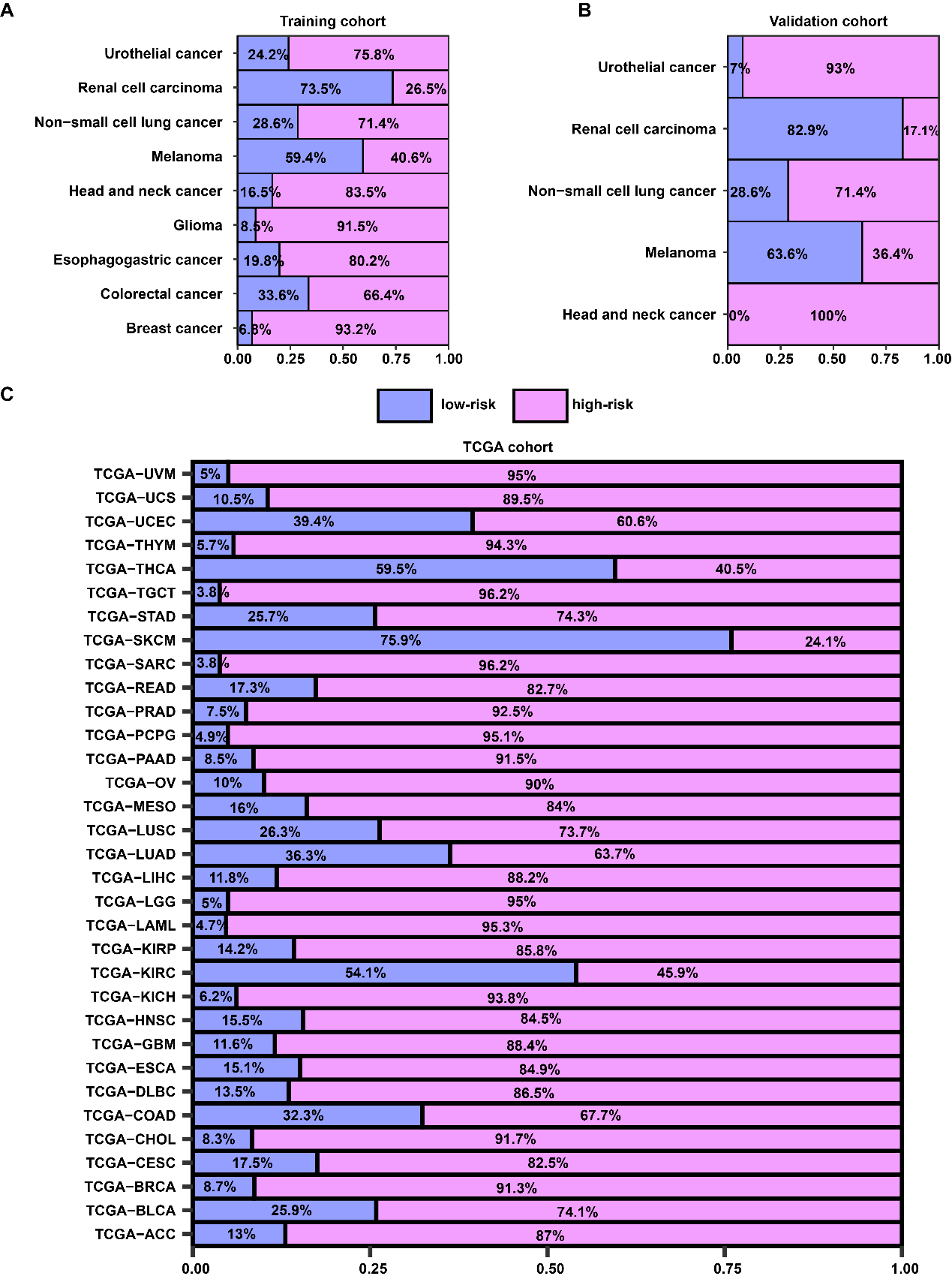


**Fig. S6: The distribution of the low-risk group and the high-risk group for each cancer type in each dataset.**

(A) The distribution of high- and low-risk groups for each cancer type in the training cohort. (B) The distribution of high- and low-risk groups for each cancer type in the validation cohort. (A) The distribution of high- and low-risk groups for each cancer type in the TCGA cohort.


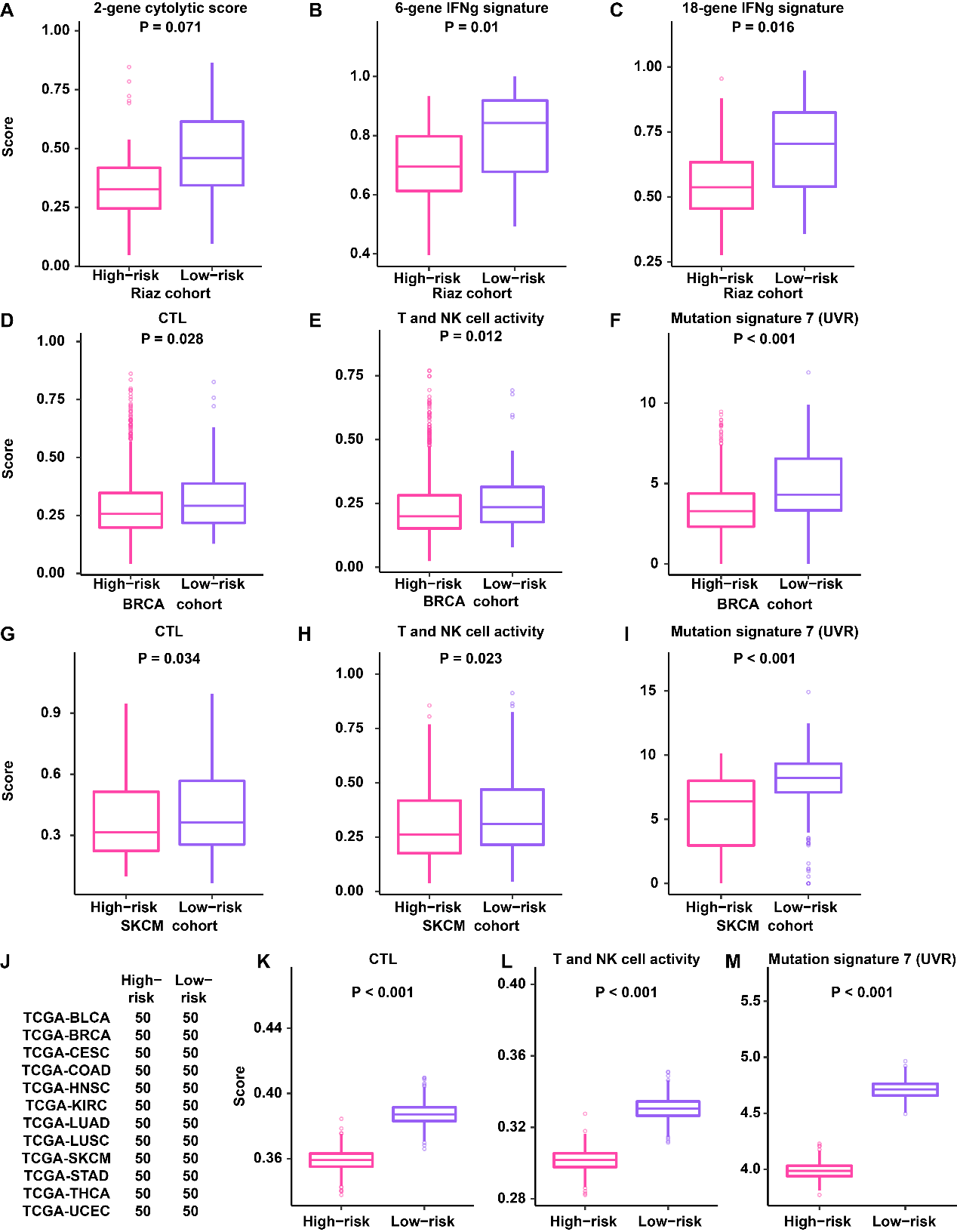


**Fig. S7: Comparison of immune activity between the low-risk group and the high-risk group.**

(A) Comparison of the 2-gene cytolytic score between the low-risk group and the high-risk group in the Riaz cohort. (B) Comparison of the 6-gene IFN-g signature score between the low-risk group and the high-risk group in the Riaz cohort. (C) Comparison of the 18-gene IFN-g signature score between the low-risk group and the high-risk group in the Riaz cohort. (D) Comparison of CTLs between the low-risk group and the high-risk group in patients with BRCA from the TCGA cohort. (E) Comparison of T and NK cell activity between the low-risk group and the high-risk group in patients with BRCA from the TCGA cohort. (F) Comparison of mutation signature 7 (UVR) between the low-risk group and the high-risk group in patients with BRCA from the TCGA cohort. (G) Comparison of CTLs between the low-risk group and the high-risk group in patients with SKCM from the TCGA cohort. (H) Comparison of T and NK cell activity between the low-risk group and the high-risk group in patients with SKCM from the TCGA cohort. (I) Comparison of mutation signature 7 (UVR) between the low-risk group and the high-risk group in patients with SKCM from the TCGA cohort. (J) Random sampling in the TCGA cohort. (K) Comparison of CTLs between the randomly sampled high-risk group and randomly sampled low-risk group in the TCGA cohort. (L) Comparison of T and NK cell activity between the randomly sampled high-risk group and randomly sampled low-risk group in the TCGA cohort. (M) Comparison of mutation signature 7 (UVR) between the randomly sampled high-risk group and randomly sampled low-risk group in the TCGA cohort.


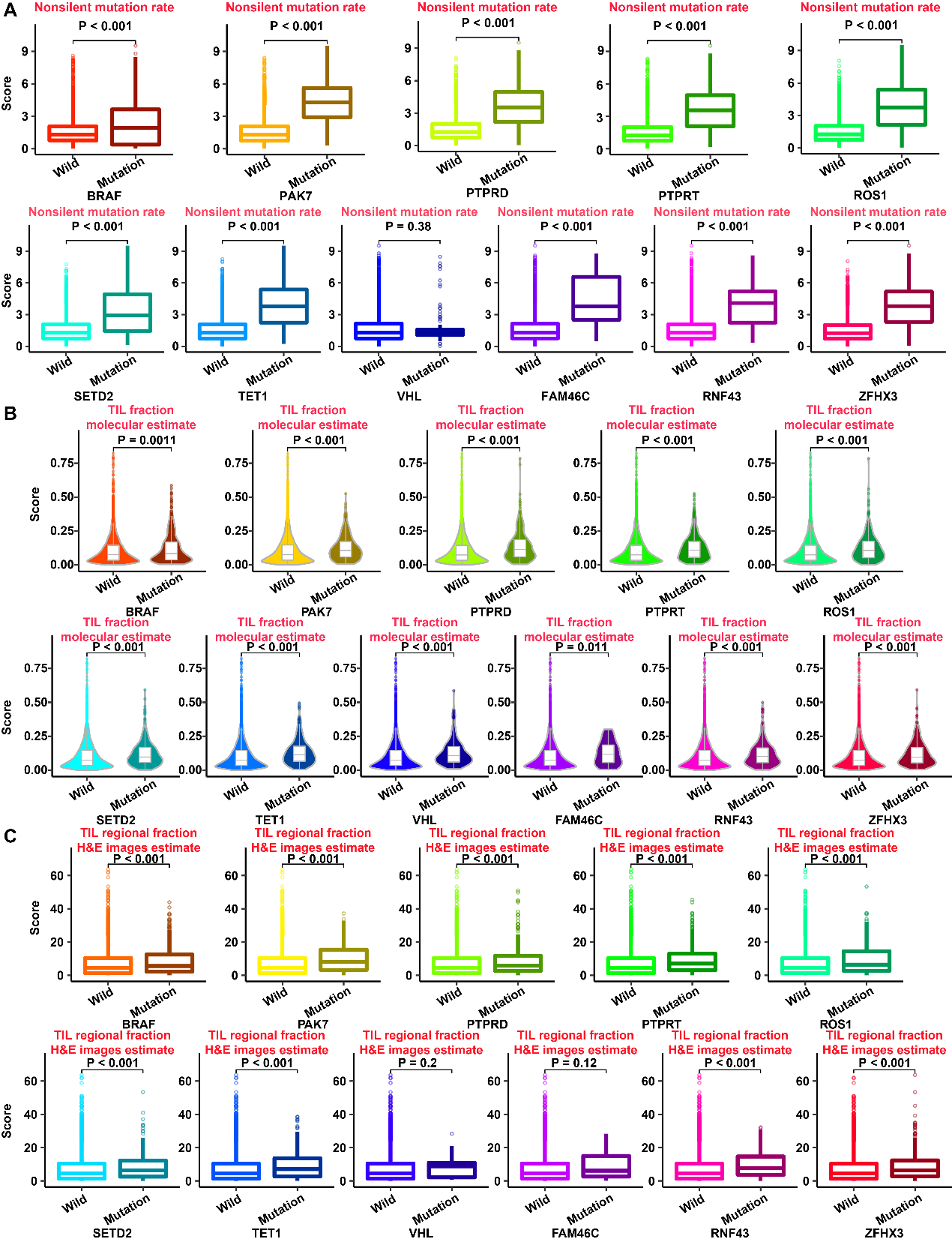


**Fig. S8: Mutations in 11 genes in the mutation-based gene set are related to enhanced tumor immunogenicity and high immune cell infiltration.**

(A) Comparison of the nonsilent mutation rate between the mutant gene and wild-type gene groups. (B) Comparison of TIL fractions based on molecular estimates from the processing of cancer genomics data between the mutant-gene and wild-type-gene groups. (C) Comparison of TIL regional fractions based on estimates from processing diagnostic H&E images between the mutant-gene and wild-type-gene groups.


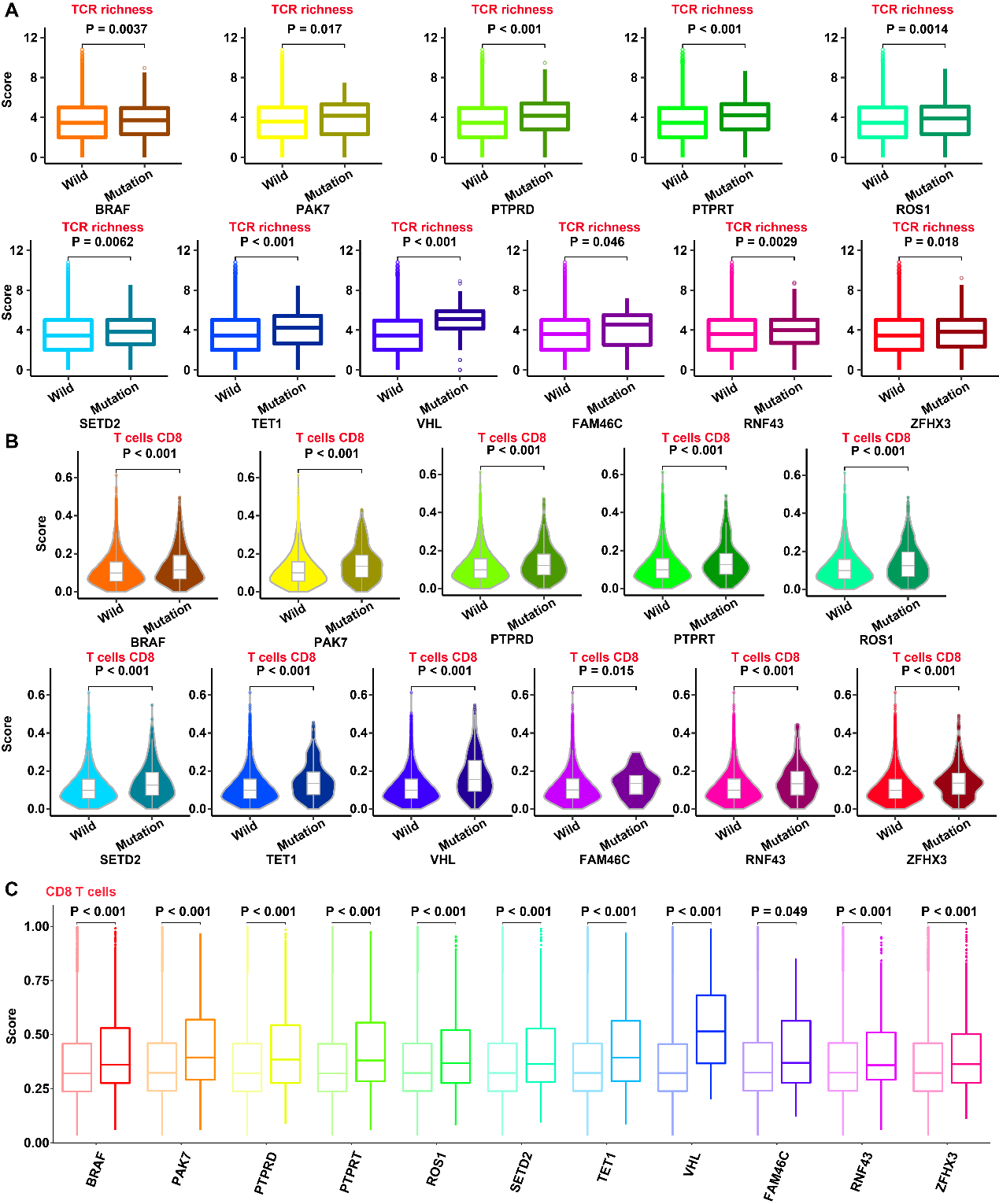


**Fig. S9: Mutations of 11 genes in the mutation-based gene set are related to high T cell infiltration.**

(A) Comparison of TCR richness between the mutant gene and wild-type gene groups. (B) Comparison of CD8 T cells estimated by the CIBERSORT method based on RNA-sequencing data between the mutant gene and wild-type gene groups. (C) Comparison of CD8 T cells estimated by the ssGSEA method based on RNA-sequencing data between the mutant gene and wild-type gene groups.


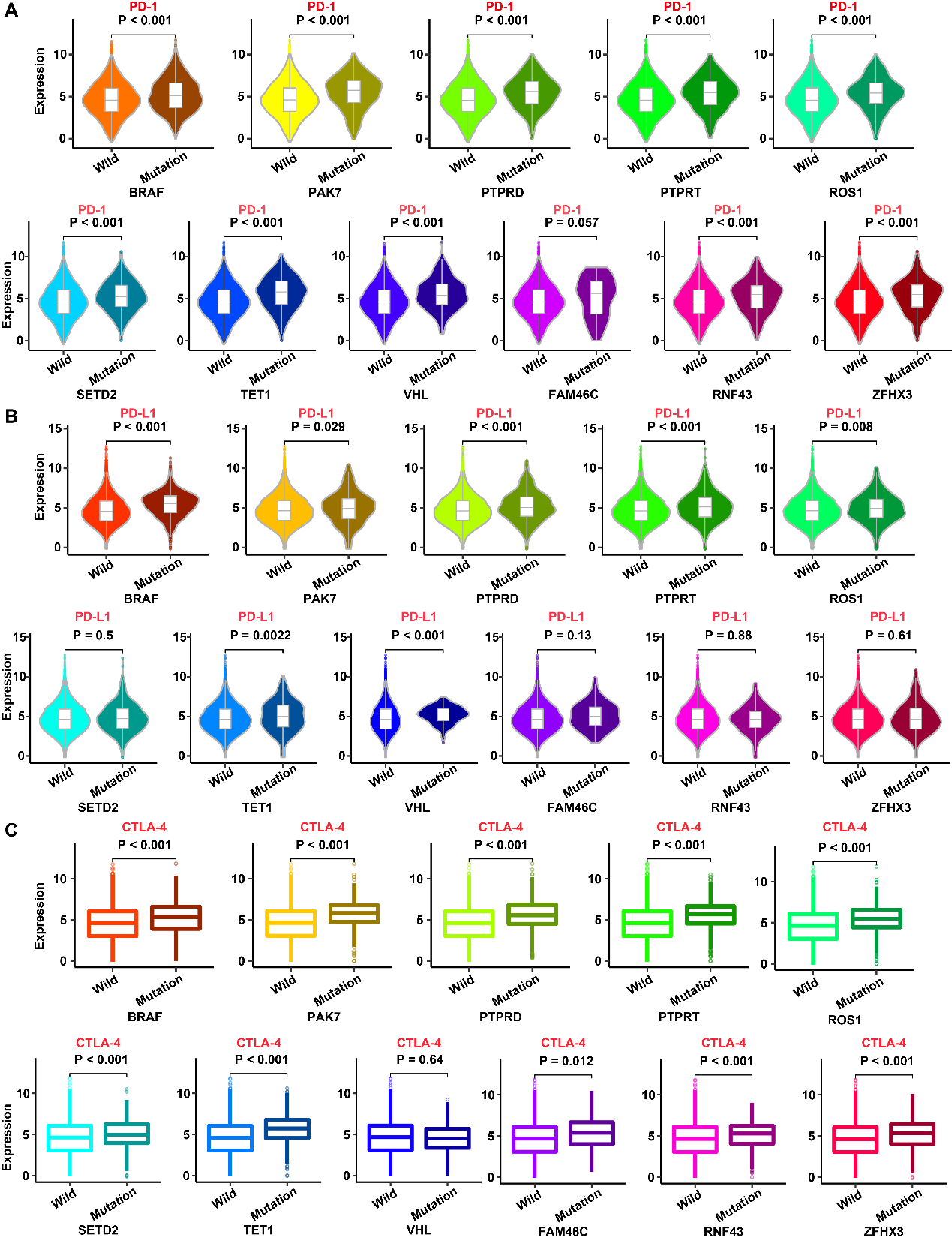


**Fig. S10: Mutations of 11 genes in the mutation-based gene set are associated with high immune checkpoint expression.**

(A) Comparison of the expression of PD-1 between the mutant gene and wild-type gene groups. (B) Comparison of the expression of PD-L1 between the mutant gene and wild-type gene groups. (C) Comparison of the expression of CTLA-4 between the mutant gene and wild-type gene groups.
